# Updated Adulticide Susceptibility Status of Florida Populations of *Aedes aegypti* (Linnaeus, 1762)

**DOI:** 10.3390/pathogens15030251

**Published:** 2026-02-27

**Authors:** Casey Parker-Crockett, Ana L. Romero-Weaver, Edwin R. Burgess, Troy J. Fedirko, Sierra M. Schluep, Leigh Ketelsen, Chelsea Dorsainvil, Natalie L. Kendziorski, Kyle J. Kosinski, Shelley A. Whitehead, Raquel Lima de Souza, Daviela Ramirez, Saul Lozano, Eva A. Buckner

**Affiliations:** 1 Centers for Disease Control and Prevention, Division of Vector-Borne Diseases, Fort Collins, CO 80521, USA; ab73@cdc.gov (C.P.-C.); nkq3@cdc.gov (S.L.); 2 Florida Medical Entomology Laboratory, Institute of Food and Agricultural Sciences, University of Florida, Vero Beach, FL 32962, USAsschluep@ufl.edu (S.M.S.);; 3 Entomology and Nematology Department, Institute of Food and Agricultural Sciences, University of Florida, Gainesville, FL 32611, USA

**Keywords:** container mosquito, insecticide resistance, pyrethroid, organophosphate, mosquito control, *Aedes aegypti*, Florida, United States

## Abstract

Insecticide resistance in *Aedes aegypti* (Linnaeus, 1762) poses a major challenge for vector control programs, undermining the effectiveness of chemical tools to mitigate both nuisance biting and the transmission of vector-borne diseases. Previous studies have documented widespread resistance to multiple adulticide active ingredients across Florida, particularly pyrethroids, along with high prevalence of knockdown resistance (*kdr*) mutations. Continued resistance monitoring is essential for guiding treatment decisions and detecting shifts in susceptibility over time. In collaboration with mosquito control programs across Florida, we assessed the susceptibility of 78 *Ae. aegypti* populations to up to six active ingredients, conducting 396 independent CDC bottle bioassays and calculating hazard ratios relative to a susceptible *Ae. aegypti* strain. For pyrethroids, 24 h post-exposure mortality was used to evaluate phenotypic recovery from knockdown. Hazard ratios revealed *Ae. aegypti* populations were more susceptible to organophosphate adulticide active ingredients, while the lowest hazard ratios, indicating higher resistance, were observed for sumithrin, deltamethrin, etofenprox, and permethrin, respectively. Evidence of knockdown resistance and recovery after 24 h was present across all pyrethroid exposures, with the highest levels following treatment with etofenprox and sumithrin. These findings confirm that pyrethroid resistance remains a significant barrier to *Ae. aegypti* control and provide updated, operationally relevant resistance data that can complement existing guidance and support evidence-based vector management strategies.

## 1. Introduction

*Aedes aegypti* (Linnaeus, 1762) is the principal vector for several arboviruses including dengue (1–4), Zika, chikungunya, and yellow fever viruses [1]. In Florida, this invasive species is widely established in the peninsular region of the state [2] and has been implicated in recent outbreaks of dengue and Zika in the state [3]. Its anthropophilic nature [4], primarily diurnal feeding activity with crepuscular peaks [5,6], and high vectorial capacity make it both a considerable nuisance and a species of public health concern. Control of *Ae. aegypti* can be challenging as the larvae occupy a wide range of container habitats that can be cryptic and/or inaccessible [7,8], making source reduction efforts challenging without well-adopted community source reduction campaigns [9,10,11]. Some wide area larviciding treatments have been effective against *Ae. aegypti* [12], but control of the adult population is still necessary to mitigate biting pressure and public health threats. Adult control via the use of adulticide ultra-low volume sprays is also difficult due to widespread insecticide resistance in *Ae. aegypti*, which has previously been characterized in Florida [13,14,15,16,17].

Currently, there are three classes of insecticides available for adult public health mosquito control in Florida: pyrethroids (IRAC Group 3A), organophosphates (IRAC Group 1B), and avermectins and milbemycins (IRAC Group 6) [18]. The latter was only recently introduced in the U.S. as part of a multi-active ingredient product and has been evaluated by some mosquito control agencies against *Ae. aegypti* with success [19,20,21]. However, guidelines for evaluating the active ingredients contained in this product in the Centers for Disease Control and Prevention (CDC) bottle bioassay have not been calibrated and released [22]. Furthermore, rotation between chemical classes remains difficult due to the limited number of chemical classes labeled for mosquito control use and because of the presence of combination products that contain multiple chemical classes in one product. Pyrethroids have been used heavily in Florida [23], and their prolonged use is compounded by the legacy use of the organochlorine (IRAC Group 3B) DDT, which acts on the same target site as pyrethroids, the voltage-gated sodium channel [18]. The documented insecticide resistance in Florida combined with limited adulticide chemical rotation options present major control challenges and threats to the protection of public health.

Globally, the trend of insecticide resistance in *Ae. aegypti* has also been observed. Insecticide resistance has been documented to all major classes of insecticides used for control, but pyrethroids have been the most prominent. Knockdown resistance (*kdr*) alleles and metabolic resistance mechanisms have been reported in *Ae. aegypti* from Asia, Africa, and the Americas [24,25,26,27]. Consequently, insecticide resistance is considered a growing challenge globally that can limit the effectiveness of mosquito control efforts, especially considering the limited chemical toolbox available for public health vector control programs. In the United States, insecticide resistance in *Ae. aegypti,* especially to pyrethroids, has been well documented, including in newly invasive populations in California [28,29,30]. This trend of insecticide resistance in *Ae. aegypti* has implications for vector control efforts and should be mitigated and monitored.

In Florida specifically, insecticide resistance in *Ae. aegypti* has been previously documented and is widespread in populations throughout the state [13,14,15]. In a statewide survey [13], 37 *Ae. aegypti* populations were tested; 95% of the assays conducted with pyrethroids resulted in resistant outcomes. In contrast, only 31.5% of assays conducted with organophosphate active ingredients resulted in resistance. Studies conducted on the intensity of the resistance detected revealed resistance ratios as high as 61 to permethrin [14], a pattern dissimilar from *Ae. albopictus* that exhibited a much lower prevalence of pyrethroid resistance [13]. Caged field trials conducted throughout the state also indicate that formulated products containing pyrethroids achieve lower mortality compared to products containing other modes of action [19,20].

With the demonstration of insecticide resistance in laboratory assays and indications of decreased efficacy in the field, the importance of monitoring for changes in susceptibility and identifying active ingredients with high prevalence of susceptibility is critical for *Ae. aegypti*. Here, we conducted a statewide insecticide resistance survey in Florida to build upon the work of Parker et al. [13]. The CDC bottle bioassay was used to test against four pyrethroid and two organophosphate active ingredients with the goal of providing insecticide resistance data to vector control programs and updating the current resistance data available in the state.

## 2. Materials and Methods

### 2.1. Mosquito Egg Collection and Rearing

From 2019 to 2022, container *Aedes* oviposition kits were provided to Florida mosquito control programs (MCPs) upon request, as previously described [13]. Kits contained seed germination paper, 355 mL black plastic cups, and binder clips and were assembled by the MCPs by attaching the seed germination paper to interior lip of the cup with the provided binder clips. After assembly, MCPs deployed the water-filled oviposition cups in the field, collecting them after 5–7 days had elapsed. Container mosquito eggs on germination paper were mailed weekly to the Florida Medical Entomology Laboratory (FMEL) in plastic bags. Upon receipt, egg papers were visually inspected, allowed to air dry, and stored for a minimum of one week before attempting to hatch the eggs. Eggs were hatched after MCPs had provided enough eggs to conduct several CDC bottle bioassays.

Once enough eggs had been collected to conduct CDC bottle bioassays, eggs were hatched at a density of 250 eggs per 40.6 × 15.4 × 6.4 cm enamel tray containing two liters of distilled water. Mosquitoes were reared in an insectary maintained at 27 °C ± 2 and 70% ± 5 relative humidity. Developing larvae were provided a 1:1-by-weight mixture of yeast and lactalbumin (MP Biomedicals LLC, Irvine, CA, USA) ad libitum. Upon pupation, mosquitoes were transferred to emergence cups using a plastic pipette. Cups containing pupae were placed in a plastic 30 × 30 × 30 cm cage (Bioquip, Rancho Dominguez, CA, USA) and provisioned with 10% sucrose solution. Approximately 7 days after pupal emergence, mosquitoes were offered bloodmeal from a live chicken (IACUC #202007682) for approximately 45 min. An oviposition cup lined with germination paper and filled partially with water was placed inside the cage after bloodfeeding. The offspring of field-collected eggs were the F1 generation, and only mosquitoes within two generations of the field-collected eggs were used in subsequent assays.

### 2.2. CDC Bottle Bioassay

The CDC bottle bioassay [22] was used to assess the insecticide susceptibility status of field-collected populations of *Ae. aegypti*. Technical-grade active ingredients obtained from ChemService (West Chester, PA, USA) and the CDC were used in the assay. Stock solutions were created for the following active ingredients with acetone: deltamethrin (0.75 µg/mL); etofenprox (12.5 µg/mL); malathion (400 µg/mL); naled (2.25 µg/mL); permethrin (43 µg/mL); sumithrin (20 µg/mL). Each time a new stock solution was made, it was calibrated using the Orlando (1952) susceptible strain of *Ae. aegypti* [31] to establish the diagnostic time, the time point we expect 100% mortality in a field population if it is susceptible to the insecticide. Each CDC bottle bioassay consisted of four 250 mL Wheaton bottles treated with 1 mL of a particular active ingredient stock solution and one 250 mL Wheaton bottle treated with 1 mL of acetone (negative control). Only one bottle bioassay replicate for each active ingredient was conducted for each field *Ae. aegypti* population. Bottles were coated by placing them on a bottle roller (Vollrath Cayenne Hot Dog Roller Grill model number 40821, Sheboygan, WI, USA with heat turned off) for five minutes with caps on followed by three minutes on the bottle roller with caps off. Following treatment, bottles were stored in a cabinet or drawer to protect from light exposure for 14 to 24 h before assays were conducted.

Approximately 20–25 mosquitoes were aspirated into each of the five bottles and mortality was recorded at 0, 5, 15, 30, 45, 60, 75, 90, 105, and 120 min after the introduction of mosquitoes into the bottle. Mosquitoes were counted as dead when they were unable to right themselves or exhibited erratic/uncoordinated flight behavior [22]. At the conclusion of the 120 min assay, mosquitoes exposed to pyrethroid active ingredients (deltamethrin, etofenprox, permethrin, and sumithrin) were transferred to clean paper cups with a mesh covering, as described in Parker [32]. For the pyrethroid active ingredients, 24 h mortality was recorded to assess phenotypic knockdown resistance (*kdr*). Abbott’s formula was used to correct mortality in the insecticide-treated bottles when mortality in the control bottle was between 3 and 10% [22,33]. If mortality in the control bottles exceeded 10%, the assay was discarded. Assays were evaluated for consistency among treated bottles. When mortality in a single treated bottle was markedly discordant from the others within the assay—for example, when 100% mortality was observed in three bottles by 30 min, but no mortality occurred in the fourth—that bottle was excluded from the replicate. Excluded bottles were not included in subsequent analyses. Following the definitions from the CDC bottle bioassay protocol [22], mosquitoes were classified as susceptible if they achieved at least 97% mortality at the diagnostic time, developing resistance if mortality was between 90 and 96%, and resistant if mortality was less than 90%.

### 2.3. Data Handling and Statistics

Twenty-four-hour recovery was used as a quantitative measurement of phenotypic knockdown resistance and calculated as in [34] by subtracting the percent mortality at 24 h from the percent mortality at the end of the assay and dividing the difference by the percent mortality at the end of the assay.

All statistical analyses were conducted in R version 4.5.0 as previously described by Burgess et al. [35]. For CDC bottle bioassays, a clustered Cox regression was generated on pairwise comparisons of each field *Ae. aegypti* population’s mortality over time to that of the susceptible Orlando (1952) *Ae. aegypti* strain using the ‘survival’ package (version 3.8-3) [36]. Separate analyses were run for deltamethrin, etofenprox, permethrin, malathion, naled, and sumithrin. Due to the presence of four treated bottles used per population, the clustering effect was assigned for each of the treatments (insecticides) to account for variability among replicates. In the deltamethrin, etofenprox, malathion, naled, permethrin, and sumithrin pairwise analyses, the field *Ae. aegypti* populations were compared to the susceptible *Ae. aegypti* ORL strain with diagnostic times of 15, 45, 30, 30, 15, and 30 min, respectively, which were the most conservative diagnostic times for all assays performed with each active ingredient.

Cox models were assessed for the assumption of proportionality of hazard over time prior to statistical testing using the ‘cox.zph()’ function. A time-dependent coefficient was added to account for the interaction between treatment and the time in minutes for models that violated this assumption. These hazard ratios (HRs) are reported with a time factor change in hazard rate of mortality of each field strain relative to the Orlando susceptible *Ae. aegypti* strain. For ties in times to death, the Efron approximation was used. A Wald test was used to test the null hypothesis that the beta coefficients = 0 at α = 0.05.

If the 95% confidence interval included 1.0 or the *p*-value was greater than 0.05, this was interpreted as insufficient evidence of a difference between the field strain and the reference strain (Orlando susceptible *Ae. aegypti* strain). A statistically significant hazard ratio value > 1.0 is interpreted as a fold increase in the hazard rate of mortality relative to the reference over the time interval, while hazard ratios < 1.0 are interpreted as a proportion of the hazard rate of mortality relative to the reference (e.g., HR = 1.5 is a 1.5-fold likelihood of mortality across the time interval, or 50% increased hazard rate of mortality, relative to the Orlando susceptible *Ae. aegypti* strain; HR = 0.7 is 70% of the likelihood of mortality over the time interval relative to the reference strain, or a 30% reduced hazard rate of mortality). Reliable hazard ratios could not be calculated for assays where less than 10% mortality was achieved by the end of the 2 h assay. These assays were not included in Cox proportional hazards analysis.

## 3. Results

A total of 396 CDC bottle bioassays were conducted representing insecticide resistance data from 78 unique *Ae. aegypti* populations from 18 counties. Detailed population level data, CDC bottle bioassay results, and HR outputs for individual assays can be found in the Supplementary Materials.

**Figure 1:**
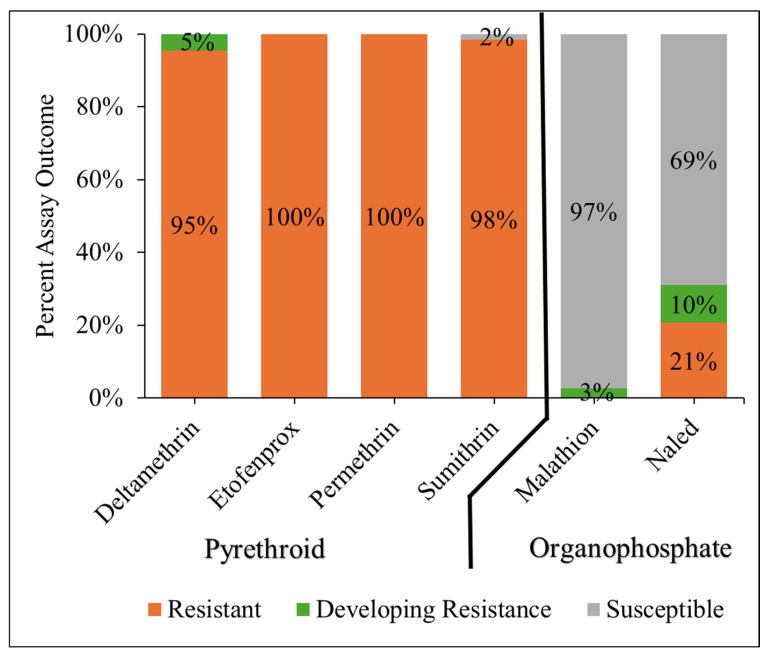
Distribution of assay outcomes for the CDC bottle bioassay for each active ingredient, grouped by chemical class. Assay outcomes were classified as resistant (orange), developing resistance (green), or susceptible (gray), based on percent mortality at the established diagnostic time for each active ingredient. Percentages reflect the proportion of total assays conducted with each active ingredient that resulted in each classification.

The resistance outcomes of each bottle bioassay (resistant, developing resistance, and susceptible) varied sharply between chemical class (Figure 1). For permethrin and etofenprox, 100% of assays conducted resulted in a resistant outcome. Nearly all assays conducted with sumithrin resulted in a resistant outcome (98%), but 2% (1 assay) were classified as susceptible, which was the only susceptible outcome for a pyrethroid assay. Deltamethrin assays resulted in 95% of assays indicating resistance and 5% indicating resistance was developing. In contrast to the pyrethroids, organophosphate active ingredients produced primarily susceptible outcomes with 97% of malathion assays resulting in a susceptible outcome and 69% of naled assays indicating the same (Figure 1). Overall, the frequency of resistant outcomes was substantially higher with pyrethroid active ingredients compared to organophosphate active ingredients.

**Figure 2:**
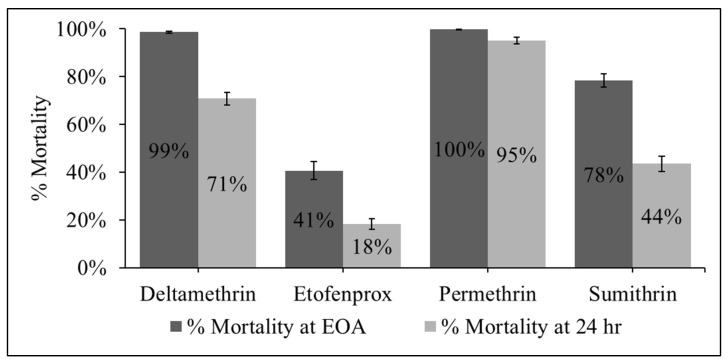
Unweighted mean percent mortality across all tested populations at the end of the CDC bottle bioassay (EOA) and at 24 h post-exposure. For each insecticide and time point, population-level mortality estimates were averaged. Error bars represent the standard error of the mean (SEM) calculated from those population-level values. A decrease in percent mortality between the EOA reading and the 24 h reading indicates recovery.

Comparisons of mortality at the end of the assay (EOA) and at 24 h were conducted for the pyrethroid active ingredients as a phenotypic indicator of *kdr*. All pyrethroid active ingredients resulted in some recovery (Figure 2). Etofenprox exhibited the steepest decline from 41% mortality at EOA to 18% at 24 h. Sumithrin similarly decreased from 78% to 44% and deltamethrin declined from 99% to 71%. The smallest reduction in mortality was observed in response to permethrin, with an average EOA mortality of 99.8% and an average 24 h mortality of 95%.

**Figure 3:**
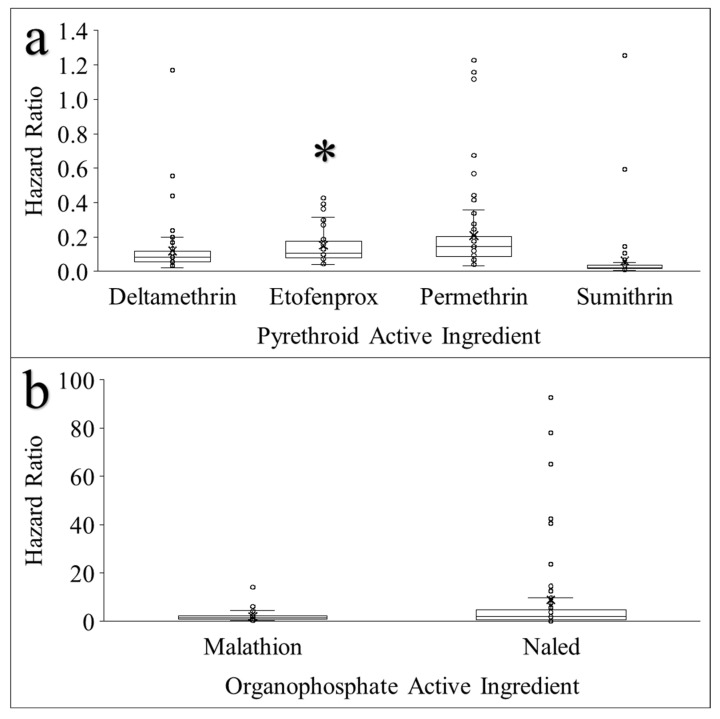
Distributions of hazard ratios for all Florida *Ae. aegypti* populations tested in response to pyrethroid (**a**) and organophosphate (**b**) active ingredients. The lower and upper hinge of boxes represent the 25th and 75th quartiles, respectively. The middle line represents the median, and the “x” within each box represents the mean. The whiskers represent the 10th and 90th percentiles. * Twelve assays conducted with etofenprox could not have reliable hazard ratios calculated due to the low mortality throughout the assay (less than 10% by the end of the 2 h assay). Note: The scale of the y-axis is different between panel (**a**,**b**).

Hazard ratios calculated for individual active ingredients and for chemical classes mirrored the trends observed in assay outcomes (Figure 1). Pyrethroids had a lower median hazard ratio (0.088) compared to organophosphates (1.508). All pyrethroid active ingredients exhibited low median hazard ratios ranging from 0.022 for sumithrin to 0.081 for deltamethrin, 0.108 for etofenprox, and 0.146 for permethrin (Figure 3a). For etofenprox, hazard ratio estimates are likely inflated due to the exclusion of 12 assays in which mortality remained below 10% at the end of the 2 h exposure period, precluding reliable estimation using Cox proportional hazards models. Inclusion of these assays, had model convergence been possible, would be expected to yield hazard ratios approaching zero and would therefore further reduce both the mean and median hazard ratio estimates for etofenprox. As a result, the reported hazard ratios for etofenprox should be interpreted as conservative estimates of its relative effect. Between the tested populations, the hazard ratios were the most variable in response to permethrin, followed by etofenprox, deltamethrin, and sumithrin (Figure 3a). The median hazard ratios for malathion (1.419) and naled (1.903) were much higher than the pyrethroid active ingredients. Greater variability in hazard ratios among the tested populations was observed for naled compared with malathion (Figure 3b).

**Figure 4:**
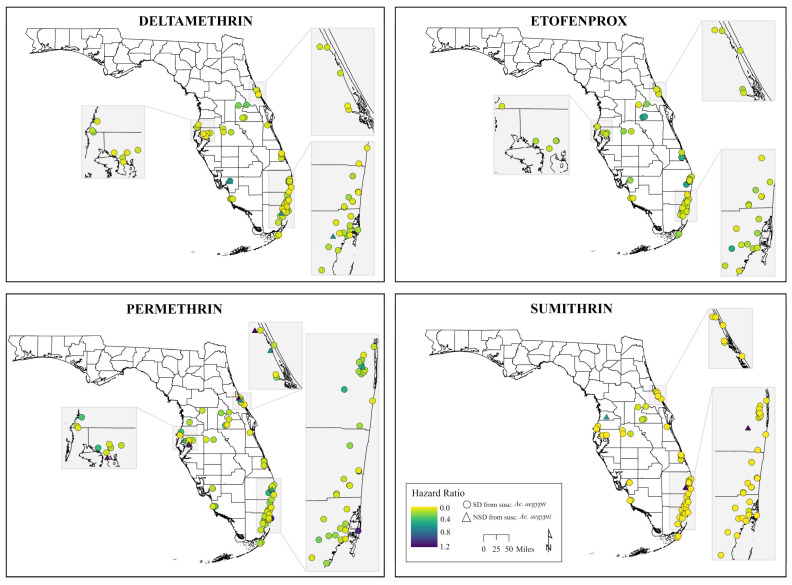
Spatial distribution of hazard ratios of field *Aedes aegypti* populations across Florida relative to the susceptible Orlando strain for the pyrethroids deltamethrin, etofenprox, permethrin, and sumithrin. Map created using ArcGIS Pro (Version 3.5.0). Hazard ratios (HRs) are presented relative to the reference population. An HR < 1 indicates greater resistance (lower hazard compared to the reference), whereas an HR > 1 indicates greater susceptibility. Thus, smaller HR values correspond to higher levels of resistance.

**Figure 5:**
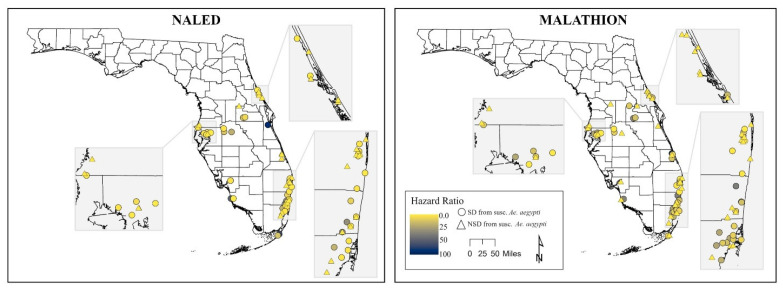
Spatial distribution of hazard ratios of field *Aedes aegypti* populations across Florida relative to the susceptible Orlando strain for the organophosphates malathion and naled. Map created using ArcGIS Pro. Hazard ratios (HRs) are presented relative to the reference population. An HR < 1 indicates greater resistance (lower hazard compared to the reference), whereas an HR > 1 indicates greater susceptibility. Thus, smaller HR values correspond to higher levels of resistance.

Figure 4 shows that most *Ae. aegypti* populations across the state have low hazard ratios relative to the susceptible strain, indicating resistance to pyrethroids is widespread. Permethrin shows the greatest variability geographically, with a small number of sites (notably along the east coast and in isolated locations) exhibiting higher hazard ratios, suggesting comparatively greater susceptibility at those locations. Deltamethrin, etofenprox, and sumithrin display more uniformly low hazard ratios statewide, with fewer populations deviating from highly resistant. Organophosphate active ingredients (Figure 5) exhibited greater spatial heterogeneity compared to pyrethroids. Malathion had more moderate hazard ratios with localized areas of higher susceptibility. In contrast, naled exhibited a much higher hazard ratio statewide, particularly in central and southeastern Florida.

## 4. Discussion

Our findings indicate that the pyrethroid resistance previously observed [13,14] has persisted in Florida *Ae. aegypti* populations several years later. In Parker, Ramirez, Thomas, and Connelly [13], 95% of assays with a pyrethroid active ingredient resulted in a resistant outcome, which is similar to our findings where 98% of pyrethroid active ingredients resulted in a resistant outcome. When it comes to organophosphate active ingredients, Parker, Ramirez, Thomas, and Connelly [13] observed that 31% of assays resulted in a resistant outcome, while we only observed 9%. Except for 3 populations, all 78 of our tested *Ae. aegypti* populations were distinct from the 32 tested in Parker, Ramirez, Thomas, and Connelly [13]. However, of the three populations that were shared between the two studies (Key Largo, Forest Hills, and Leslie), changes in susceptibility status were only observed in response to malathion and naled. This aligns with the statewide trends observed in the current paper and may indicate a reversion to susceptibility for malathion in several *Ae. aegypti* populations.

Notably, many field *Ae. aegypti* populations exhibited hazard ratios greater than one for malathion and naled when compared to the insecticide susceptible ORL reference strain, indicating greater susceptibility to these active ingredients. However, hazard ratios greater than one should not be interpreted as super-susceptibility but rather higher hazard relative to susceptible ORL under these conditions. This finding highlights an important consideration for resistance interpretation, as susceptible reference strains used in bioassays may vary across laboratories and geographically due to differences in genetic background or strain being used, colony age, rearing conditions, or inadvertent selection pressures over time. Variability in susceptibility in commonly used reference strains has been documented previously and can influence study results [37,38]. This underscores the importance of the relationship between a laboratory reference strain and field strain of mosquitoes and how it influences our interpretation of insecticide resistance in that population.

Insecticide use patterns in Florida, both in mosquito control and in other industries, may be driving these resistance trends. Lloyd et al. [23] indicated that in 2014, over 90% of ground adulticide spraying was being conducted with a permethrin-based product. A more recent survey of Florida mosquito control programs revealed that a majority of respondent programs are still using pyrethroid-based products (65%), but 35% were using malathion, and 15% were using naled [39]. While these surveys are not directly comparable, the shift in the active ingredient usage signals that product rotation is occurring at several programs throughout the state. Our results support rotation to organophosphate active ingredients in most cases as 84% of the assays resulted in a susceptible outcome to organophosphates.

Rotation is a core strategy for preventing and combatting insecticide resistance, but in *Ae. aegypti*, reversion to pyrethroid susceptibility takes long periods of time to achieve. Despite a potential reduction in pyrethroid usage, we have not observed a change in the frequency of pyrethroid-resistant populations in Florida. This is supported by a field study conducted in Pasco County, FL, where only genotypic changes were observed after 2.5 years of organophosphate-only use [40]. In Mexico, cessation of pyrethroids after over 15 years of use and rotation to organophosphates led to a decrease in the lethal concentration needed to kill 50% of mosquitoes (LC_50_) and the frequency of *kdr* alleles after 6 years, but both increased shortly following the use of a multi-AI product containing a pyrethroid [41]. The resurgence of resistance in this scenario demonstrated that the use of a pyrethroid, even in combination with another active ingredient, acts as a strong selection pressure for *kdr* alleles. This resonates closely with the observations in Florida and underlines the need for a long-term commitment to insecticide resistance monitoring and rotation to meaningfully change the resistance profile of *Ae. aegypti*.

However, these studies also clearly highlight the strong selection pressure on resistant alleles in *Ae. aegypti*. In much of the Western Hemisphere, two *kdr* mutations, V1016I and F1534C, can occur in various combinations and are strongly linked to resistant phenotypes in *Ae. aegypti*, which also led to operational failure of permethrin-treated uniforms [42]. The independent rise in identical *kdr* alleles in *Ae. aegypti* populations around the world highlight the strong convergent selection on this resistance mechanism [43].

The strong and persistent pyrethroid resistance in Florida does not appear to be anomalous in this regard. In the presented study, we see this represented phenotypically in the recovery of tested populations after pyrethroid exposure. While *kdr* mutations reduce binding to the voltage-gated sodium channel [14], metabolic resistance mechanisms may further enhance survival by increasing breakdown of the insecticide [15]. Operationally, *kdr* resistance in the field may result in a temporary knockdown of host-seeking mosquitoes but not sustained lethality.

One methodological limitation of this study is that Cox proportional hazards models could not be fit for a subset of etofenprox assays due to extremely low mortality (<10%) at the conclusion of the standard 2 h CDC bottle bioassay. Although extending assay duration could potentially allow estimation of hazard ratios, this would fall outside current CDC bottle bioassay recommendations [22]. The CDC bottle bioassay’s categorical resistance classification, based on mortality at a diagnostic time point, remains interpretable under conditions of low or no mortality. Thus, while hazard ratios provide greater quantitative resolution, categorical endpoints may be more operationally useful when resistance levels are extreme.

In the presented study, variation among bottles within the same assay replicate was observed despite standardized methodology. Similar within-assay variability has been reported previously and may result from uneven insecticide coating, differences in sex composition (with males exhibiting greater susceptibility), variability in insecticide stock solutions, or environmental conditions during the assay [44,45,46]. Currently, the CDC bottle bioassay provides no formal guidance for inclusion and exclusion criteria with treated bottles when clear deviations occur. Future work could focus on the development of standardized quality control metrics or exclusion criteria that would improve assay reliability, reproducibility, and interpretability across laboratories.

The pyrethroid resistance of *Ae. aegypti* in Florida is strong and persistent, but the utilization of integrated vector management, including some novel control strategies, may provide relief from insecticide selection pressures. For example, in an *Ae. aegypti* populations exposed for 30 generations to *Bacillus thuringiensis* var. *israelensis* (*Bti*), resistance did not develop [47], and to date there is no documentation of a natural population of mosquitoes with resistance to *Bti*. A recently introduced adulticide has been shown to be effective against pyrethroid-resistant strains of *Ae. aegypti* [19,20], but it is important to note there is still pyrethroid selection pressure, and it should be rotated with a different numbered chemical class [48]. Novel non-chemical control tools including the Sterile Insect Technique (SIT) and Incompatible Insect Technique (IIT) can be implemented specifically against *Ae. aegypti* and can also be used in tandem with chemical control as necessary [49]. However, the impact of these techniques on reversion to insecticide susceptibility has not been evaluated.

Insecticide resistance in *Ae. aegypti* populations is an ongoing challenge in Florida and globally. The increase in vector control programs reporting insecticide resistance as a core competency from 2017 (14%) to 2023 (26%) [50] suggests more programs are monitoring insecticide resistance in their local populations and ideally using that information to inform vector control decisions. This was echoed in the Florida mosquito control program survey where 20 of 34 responding programs insecticide resistance was a key research priority area [39]. Future research should continue to focus on 1) documenting insecticide resistance status at the local level, 2) centrally collecting this information, and 3) investigating effective strategies to overcome resistance and revert to a susceptible state. Statewide surveys such as the presented study represent an immense effort but provide mosquito control programs with the information needed to make data-driven decisions that protect public health. Additionally, future research should focus on alternative *Ae. aegypti* control strategies, such as SIT, IIT, and entomopathogenic fungi.

## Data Availability

The original contributions presented in this study are included in this article/Supplementary Materials. Further inquiries can be directed to the corresponding author.

## Supplementary Materials

The following supporting information can be downloaded at https://www.mdpi.com/article/10.3390/pathogens15030251/s1, File S1: Master dataset (Excel); File S2: Cox regression outputs for all active ingredients (pdf).
