# Supplementary material for: Updated Adulticide Susceptibility Status of Florida Populations of Aedes aegypti (Linnaeus, 1762)
Source: Pathogens. 2026 Feb 27;15(3):251. doi: 10.3390/pathogens15030251 (PMC13029742; doi:10.3390/pathogens15030251)

Supplemental material File S2: Cox regression outputs for all active ingredients

Updated Adulticide Susceptibility Status of Florida Populations of *Aedes aegypti* (Linnaeus)

Table of contents

Each active ingredient has its own section with the following content:

1. A table of the Cox proportional hazards model output comparing the field strains to the reference strain.
2. A forest plot showing the hazard ratios and confidence intervals.
3. A Kaplan-Meier plot showing the survical curves for selected field strains and the reference strain.

Cox PH Report for Each Active Ingredient

Deltamethrin

Cox PH Regression Table

Term	log(HR)	HR	SE	z	p-value
Brevard_Viera	-2.677	0.069	0.283	-9.47	0.000
Broward_11th_St.	-3.342	0.035	0.290	-11.51	0.000
Broward_34th_Ave	-2.893	0.055	0.275	-10.52	0.000
Broward_8th_St.	-2.235	0.107	0.289	-7.72	0.000
Broward_Farragut	-2.452	0.086	0.273	-8.97	0.000
Broward_Ixtaso's_house	-2.035	0.131	0.285	-7.14	0.000
Broward_Shalimar	-2.427	0.088	0.305	-7.96	0.000
Broward_SR-7	-1.901	0.149	0.286	-6.65	0.000

Term	log(HR)	HR	SE	z	p-value
Broward_Tarpon_2020	-1.793	0.166	0.289	-6.21	0.000
Broward_Tarpon_2022	-2.300	0.100	0.279	-8.25	0.000
Collier_5th_Ave	-2.377	0.093	0.306	-7.76	0.000
Collier_Corporate_Square	-2.434	0.088	0.275	-8.85	0.000
Collier_GCC	-2.871	0.057	0.306	-9.38	0.000
Hillsborough_North_Street	-3.099	0.045	0.304	-10.19	0.000
Hillsborough_Zone_A	-3.382	0.034	0.304	-11.13	0.000
Hillsborough_Zone_B	-1.441	0.237	0.295	-4.88	0.000
Hillsborough_Zone_D	-3.239	0.039	0.334	-9.68	0.000
Hillsborough_Zone_E	-2.339	0.096	0.298	-7.85	0.000
Hillsborough_Zone_F	-2.553	0.078	0.308	-8.28	0.000
Lake_Sorrento	-1.609	0.200	0.290	-5.55	0.000
Lee_Golden_Lake	-0.824	0.439	0.285	-2.89	0.004
Lee_Luckett	0.156	1.169	0.330	0.47	0.635
MD_395	-3.095	0.045	0.298	-10.40	0.000
MD_Brickell	-3.221	0.040	0.286	-11.26	0.000
MD_Camillus_House	-2.148	0.117	0.296	-7.26	0.000
MD_Flagler_Cemetery	-3.314	0.036	0.278	-11.92	0.000
MD_Hialeah	-3.123	0.044	0.321	-9.73	0.000
MD_Homestead	-2.464	0.085	0.278	-8.85	0.000
MD_Kings_Bay	-2.356	0.095	0.278	-8.48	0.000
MD_Little_Havana	-1.669	0.188	0.279	-5.99	0.000
MD_Little_River	-2.216	0.109	0.274	-8.09	0.000
MD_Miami_Beach	-1.990	0.137	0.276	-7.22	0.000
MD_Richmond_Heights	-2.142	0.117	0.295	-7.27	0.000
MD_Westchester	-0.591	0.554	0.284	-2.08	0.038
Miami-Dade_Wynwood	-2.182	0.113	0.273	-8.00	0.000
Monroe_Key_Largo	-2.571	0.076	0.279	-9.21	0.000
Monroe_Overseas	-2.861	0.057	0.289	-9.89	0.000
Orange_Lancaster	-1.974	0.139	0.293	-6.73	0.000
Orange_Mandarin	-2.625	0.072	0.287	-9.16	0.000
Pasco_Candice	-2.900	0.055	0.282	-10.29	0.000
Pasco_Pleasure	-2.274	0.103	0.282	-8.06	0.000
PB_Andrews	-3.357	0.035	0.291	-11.53	0.000
PB_Biscayne	-2.508	0.081	0.293	-8.56	0.000
PB_Forest_Ave	-2.681	0.069	0.303	-8.84	0.000
PB_Forest_Lane	-2.596	0.075	0.300	-8.65	0.000
PB_Gardenia	-2.545	0.078	0.289	-8.82	0.000
PB_Gun_Club	-2.418	0.089	0.290	-8.35	0.000
PB_Lakewood	-2.603	0.074	0.288	-9.05	0.000
PB_Mounts_2020	-2.524	0.080	0.272	-9.26	0.000

Term	log(HR)	HR	SE	z	p-value
PB_Parkside	-3.419	0.033	0.279	-12.25	0.000
PB_Winged_Foot	-1.609	0.200	0.280	-5.74	0.000
Pinellas_Sawgrass	-1.671	0.188	0.277	-6.03	0.000
Polk_42nd_Street	-2.098	0.123	0.292	-7.17	0.000
Polk_Cheyenne_Lane	-2.483	0.084	0.313	-7.94	0.000
Polk_Wabash	-2.799	0.061	0.300	-9.34	0.000
Seminole_Halsey	-1.425	0.241	0.286	-4.99	0.000
SL_Bettys_House	-2.240	0.107	0.283	-7.91	0.000
SL_Heathcote	-2.861	0.057	0.297	-9.64	0.000
SL_Sean's_House	-2.604	0.074	0.285	-9.15	0.000
SL_Tire_Shop	-3.447	0.032	0.300	-11.51	0.000
Volusia_Holly_Hill	-2.723	0.066	0.275	-9.89	0.000
Volusia_Leslie	-3.005	0.050	0.285	-10.53	0.000
Volusia_Nova	-3.903	0.020	0.281	-13.88	0.000
Volusia_South_Daytona	-2.550	0.078	0.303	-8.40	0.000
Volusia_YMCA	-2.576	0.076	0.274	-9.41	0.000

Cox PH Forest Plot

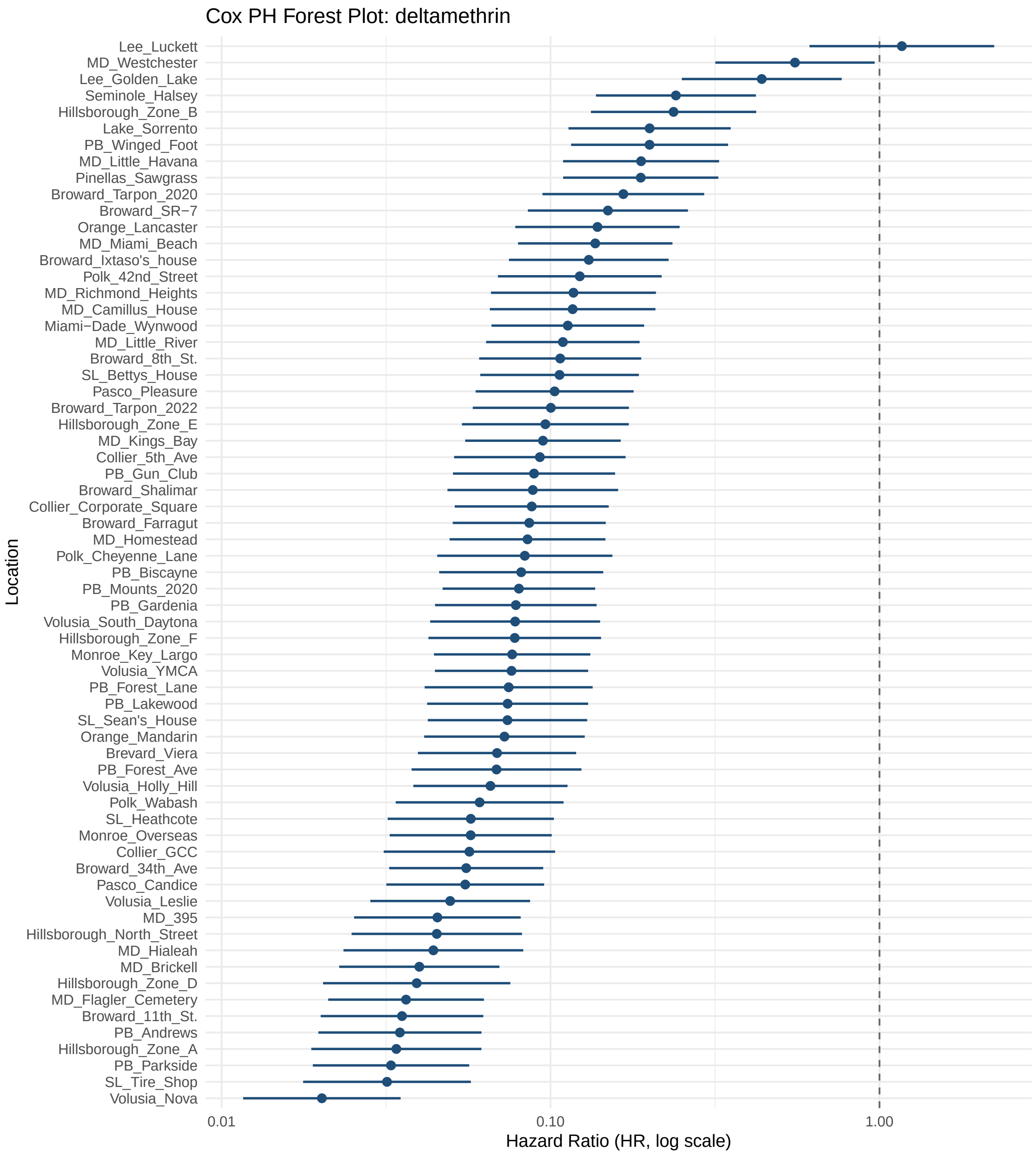

Kaplan-Meier Plot

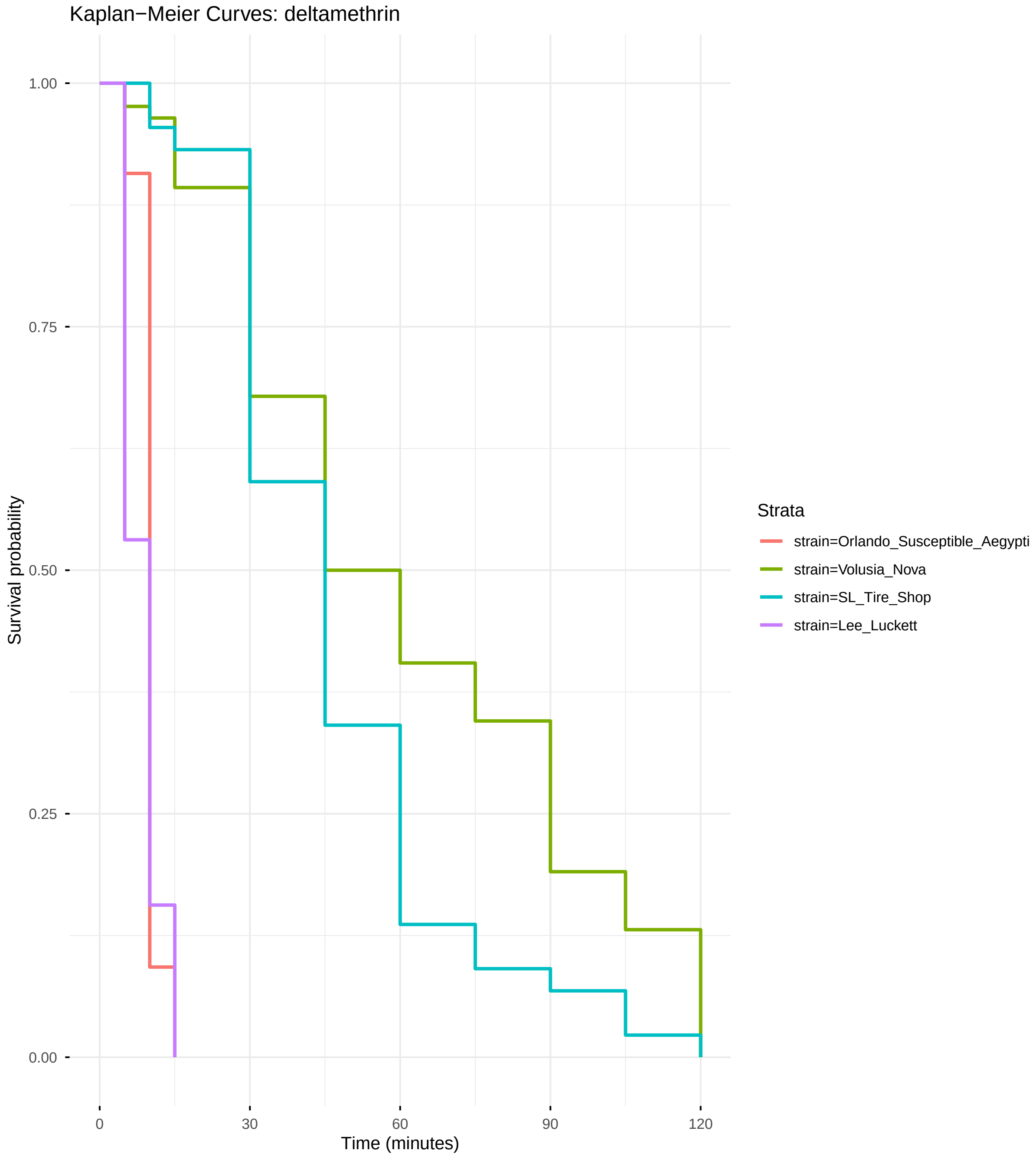

Etofenprox

Cox PH Regression Table

Term	log(HR)	HR	SE	z	p-value
Broward_34th_Ave	-2.823	0.059	0.339	-8.32	0.000
Broward_8th_St.	-2.463	0.085	0.360	-6.85	0.000
Broward_Farragut	-2.522	0.080	0.348	-7.25	0.000
Broward_Ixtaso's_house	-3.244	0.039	0.292	-11.12	0.000
Broward_Shalimar	-2.316	0.099	0.317	-7.30	0.000
Broward_SR-7	-1.830	0.160	0.348	-5.25	0.000
Broward_Tarpon_2020	-2.369	0.094	0.342	-6.92	0.000
Collier_5th_Ave	-2.295	0.101	0.323	-7.11	0.000
Collier_GCC	-3.092	0.045	0.434	-7.13	0.000
Hillsborough_North_Street	-1.211	0.298	0.320	-3.79	0.000
Hillsborough_Zone_A	-2.124	0.120	0.326	-6.52	0.000
Hillsborough_Zone_B	-2.005	0.135	0.401	-5.00	0.000
Hillsborough_Zone_E	-2.386	0.092	0.431	-5.54	0.000
Lake_Sorrento	-3.030	0.048	0.422	-7.18	0.000
Lee_Golden_Lake	-1.841	0.159	0.294	-6.26	0.000
Lee_Luckett	-1.595	0.203	0.299	-5.33	0.000
MD_395	-2.254	0.105	0.402	-5.61	0.000
MD_Camillus_House	-1.984	0.138	0.291	-6.81	0.000
MD_Flagler_Cemetery	-2.576	0.076	0.295	-8.73	0.000
MD_Hialeah	-3.059	0.047	0.375	-8.15	0.000
MD_Kings_Bay	-2.826	0.059	0.344	-8.22	0.000
MD_Little_Havana	-2.228	0.108	0.311	-7.16	0.000
MD_Little_River	-1.846	0.158	0.283	-6.51	0.000
MD_Richmond_Heights	-2.229	0.108	0.560	-3.98	0.000
MD_Westchester	-0.938	0.391	0.312	-3.00	0.003
Miami-Dade_Brickell	-2.586	0.075	0.319	-8.10	0.000
Monroe_Key_Largo	-1.770	0.170	0.288	-6.14	0.000
Monroe_Overseas	-1.796	0.166	0.299	-6.01	0.000
Orange_Lancaster	-0.939	0.391	0.314	-2.99	0.003
Orange_Mandarin	-0.852	0.427	0.305	-2.79	0.005
Pasco_Pleasure	-2.735	0.065	0.283	-9.65	0.000
PB_Flager	-2.127	0.119	0.413	-5.14	0.000
PB_Forest_Ave	-2.812	0.060	0.300	-9.38	0.000
PB_Forest_Lane	-1.162	0.313	0.321	-3.62	0.000
PB_Gardenia	-1.310	0.270	0.370	-3.54	0.000
PB_Lakewood	-2.140	0.118	0.333	-6.43	0.000

Term	log(HR)	HR	SE	z	p-value
PB_Mounts_2020	-2.446	0.087	0.361	-6.79	0.000
PB_Ranch_Dr	-1.000	0.368	0.363	-2.75	0.006
PB_US_441	-0.822	0.440	0.332	-2.47	0.013
PB_Winged_Foot	-1.764	0.171	0.369	-4.78	0.000
Polk_42nd_Street	-2.272	0.103	0.443	-5.13	0.000
Polk_Wabash	-1.680	0.186	0.527	-3.19	0.001
Seminole_Halsey	-1.678	0.187	0.306	-5.48	0.000
SL_Sean's_House	-1.014	0.363	0.326	-3.11	0.002
SL_Tire_Shop	-2.374	0.093	0.479	-4.96	0.000
Volusia_Holly_Hill	-3.097	0.045	0.310	-10.00	0.000
Volusia_Leslie	-2.617	0.073	0.364	-7.19	0.000
Volusia_Nova	-2.056	0.128	0.357	-5.76	0.000
Volusia_S._Pennisula	-2.430	0.088	0.380	-6.39	0.000
Volusia_YMCA	-2.365	0.094	0.305	-7.75	0.000

Cox PH Forest Plot

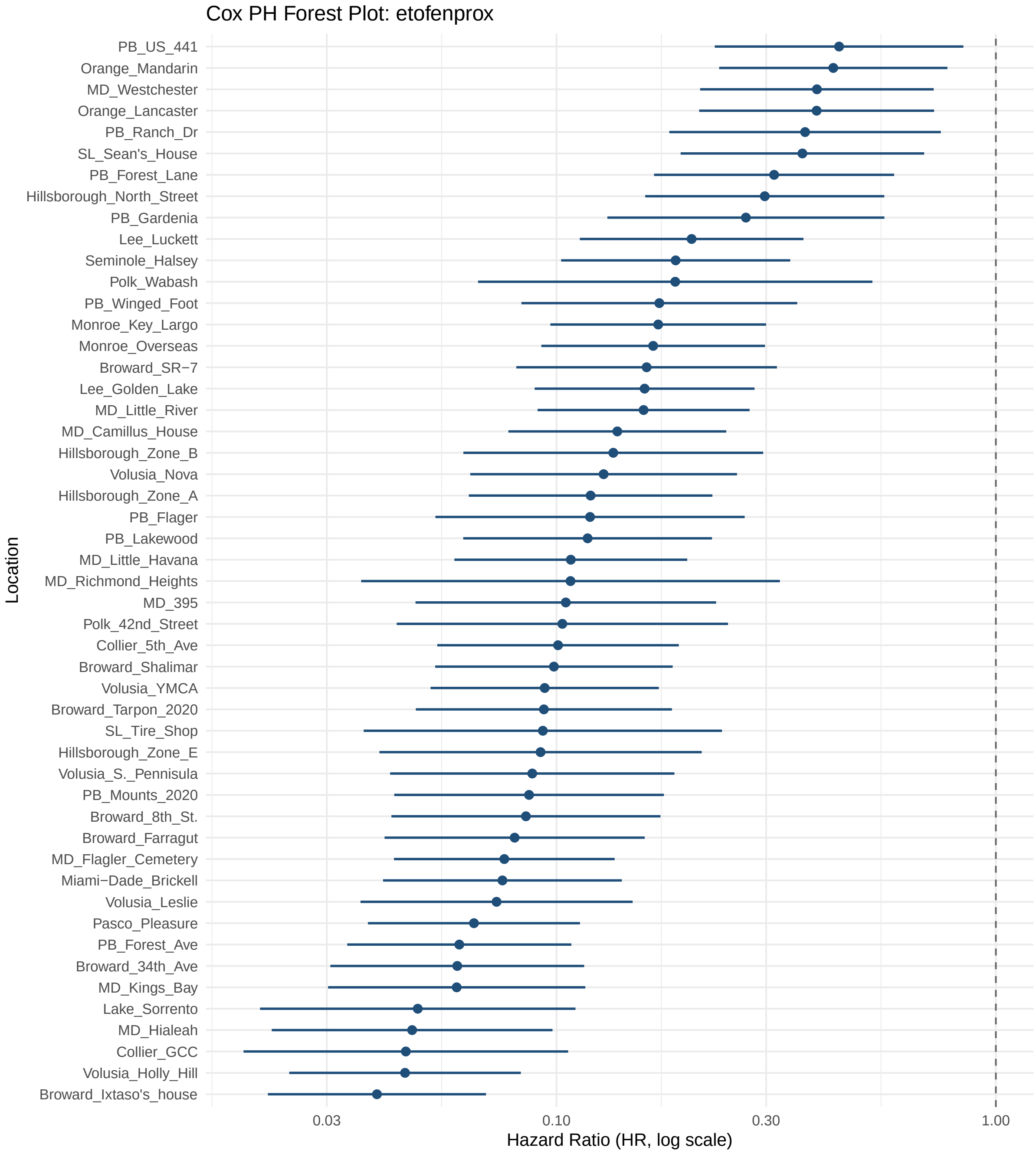

Kaplan-Meier Plot

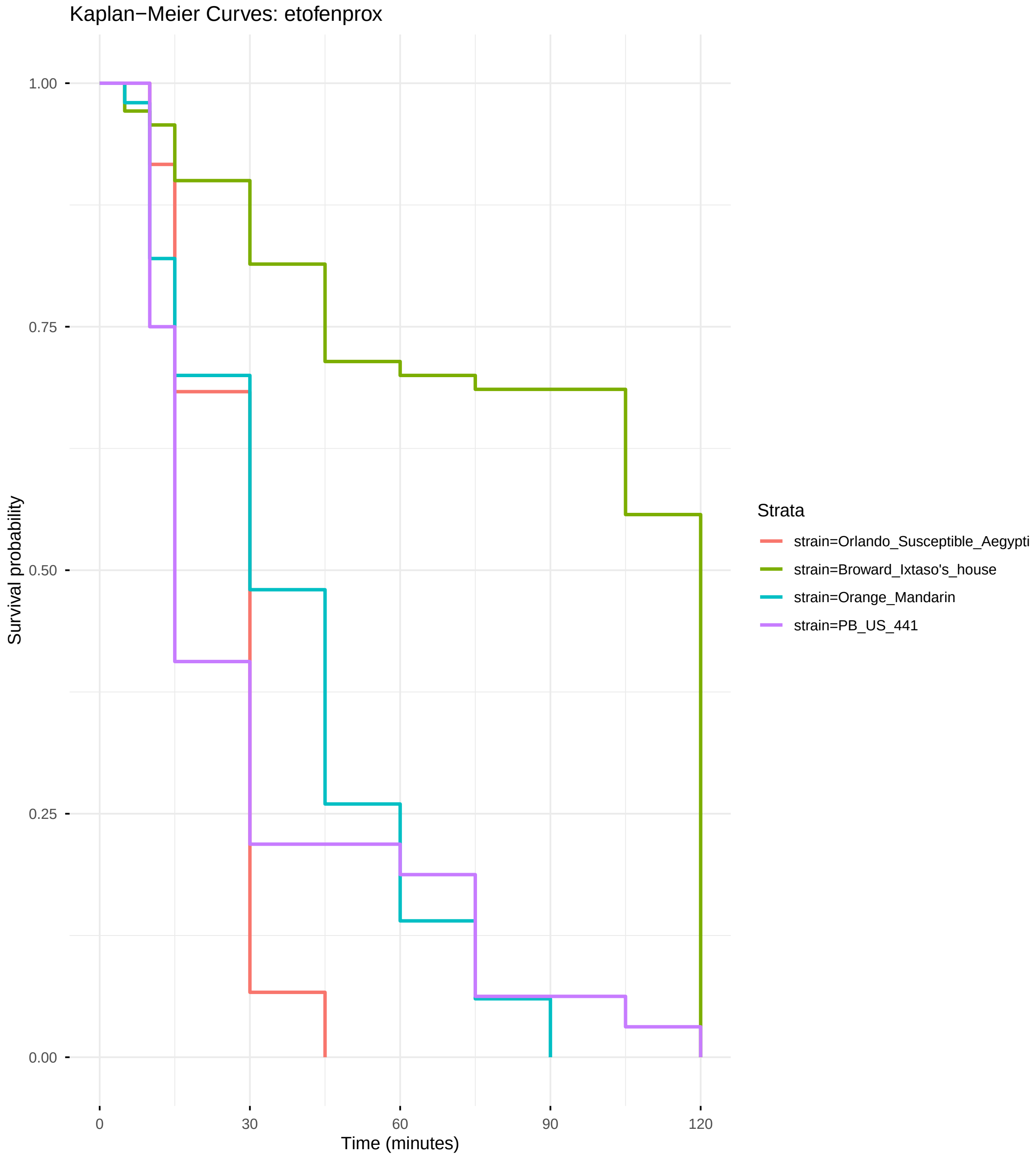

Malathion

Cox PH Regression Table

Term	log(HR)	HR	SE	z	p-value
Brevard_Viera	0.205	1.227	0.243	0.84	0.399
Broward_11th_Ave	1.366	3.921	0.256	5.34	0.000
Broward_11th_St.	1.047	2.848	0.254	4.11	0.000
Broward_34th_Ave	0.377	1.458	0.244	1.55	0.122
Broward_8th_St.	1.274	3.575	0.249	5.12	0.000
Broward_Farragut	0.669	1.952	0.236	2.83	0.005
Broward_Ixtaso's_house	1.976	7.211	0.244	8.08	0.000
Broward_Shalimar	-0.180	0.836	0.257	-0.70	0.484
Broward_SR-7	0.167	1.181	0.246	0.68	0.499
Broward_Tarpon_2020	0.233	1.262	0.247	0.94	0.345
Collier_5th_Ave	0.439	1.551	0.254	1.73	0.084
Collier_GCC	1.922	6.836	0.243	7.92	0.000
Hillsborough_North_Street	0.679	1.972	0.257	2.64	0.008
Hillsborough_Zone_A	1.469	4.345	0.242	6.08	0.000
Hillsborough_Zone_B	0.338	1.402	0.258	1.31	0.191
Hillsborough_Zone_E	-0.644	0.525	0.271	-2.38	0.017
Hillsborough_Zone_F	-0.752	0.472	0.272	-2.77	0.006
Hilsborough_Forest_Hills	0.995	2.705	0.287	3.47	0.001
Lake_Sorrento	0.755	2.128	0.243	3.11	0.002
Lee_Golden_Lake	-0.066	0.936	0.246	-0.27	0.789
Lee_Luckett	0.285	1.330	0.246	1.16	0.246
MD_395	0.631	1.880	0.242	2.61	0.009
MD_Allapattah	1.438	4.212	0.281	5.12	0.000
MD_Camillus_House	1.041	2.832	0.261	3.99	0.000
MD_Flagler_Cemetery	0.765	2.148	0.236	3.24	0.001
MD_Hialeah	1.492	4.446	0.244	6.10	0.000
MD_Homestead	0.151	1.163	0.240	0.63	0.530
MD_Kings_Bay	1.332	3.789	0.241	5.53	0.000
MD_Little_Havana	0.411	1.508	0.236	1.74	0.082
MD_Little_River	0.560	1.751	0.244	2.30	0.021
MD_Miami_Beach	0.505	1.657	0.233	2.17	0.030
MD_Richmond_Heights	-0.167	0.846	0.243	-0.69	0.491
MD_Westchester	1.503	4.496	0.241	6.23	0.000
MD_Wynwood	0.589	1.803	0.237	2.49	0.013
Miami-Dade_Brickell	0.239	1.270	0.237	1.01	0.313
Monroe_Key_Largo	0.572	1.772	0.256	2.24	0.025

Term	log(HR)	HR	SE	z	p-value
Monroe_Overseas	0.228	1.256	0.241	0.94	0.345
Orange_Lancaster	1.016	2.763	0.263	3.86	0.000
Orange_Mandarin	1.828	6.224	0.242	7.55	0.000
Pasco_Candice	-0.236	0.790	0.274	-0.86	0.389
Pasco_Pleasure	-0.051	0.950	0.245	-0.21	0.835
PB_Andrews	0.003	1.003	0.249	0.01	0.989
PB_Biscayne	-0.010	0.990	0.242	-0.04	0.968
PB_Flager	-0.056	0.946	0.252	-0.22	0.825
PB_Forest_Ave	-0.467	0.627	0.308	-1.52	0.129
PB_Forest_Lane	-0.366	0.693	0.233	-1.57	0.116
PB_Gardenia	-0.915	0.401	0.249	-3.67	0.000
PB_Gun_Club	0.148	1.159	0.270	0.55	0.584
PB_Lakewood_Ave	0.036	1.037	0.254	0.14	0.887
PB_Mounts_2020	0.670	1.955	0.249	2.69	0.007
PB_Parkside	-0.489	0.613	0.243	-2.02	0.044
PB_Ranch_Dr	1.454	4.282	0.236	6.16	0.000
PB_US_441	-0.492	0.611	0.245	-2.01	0.044
PB_Winged_Foot	0.201	1.223	0.249	0.81	0.419
Pinellas_Sawgrass	0.795	2.215	0.233	3.42	0.001
Polk_42nd_Street	-0.134	0.875	0.247	-0.54	0.589
Polk_Cheyenne_Lane	-0.606	0.545	0.255	-2.38	0.017
Polk_Wabash	0.636	1.889	0.274	2.32	0.020
Seminole_Halsey	0.363	1.437	0.235	1.54	0.124
SL_Bettys_House	0.000	1.000	0.241	0.00	1.000
SL_Heathcote	0.582	1.790	0.249	2.34	0.019
SL_Sean's_House	0.907	2.476	0.258	3.51	0.000
SL_Tire_Shop	2.652	14.187	0.261	10.17	0.000
Sumter_Tommy's_tire	0.271	1.311	0.287	0.94	0.346
Volusia_Holly_Hill	0.251	1.286	0.233	1.08	0.281
Volusia_Leslie	-0.460	0.632	0.248	-1.85	0.064
Volusia_N._Pine	1.172	3.228	0.245	4.78	0.000
Volusia_Nova	-0.426	0.653	0.241	-1.77	0.077
Volusia_Pennisula	0.257	1.294	0.251	1.03	0.305
Volusia_S._Pine	0.396	1.486	0.241	1.64	0.100
Volusia_South_Daytona	-0.332	0.717	0.249	-1.33	0.183
Volusia_YMCA	0.049	1.050	0.237	0.21	0.837

Cox PH Forest Plot

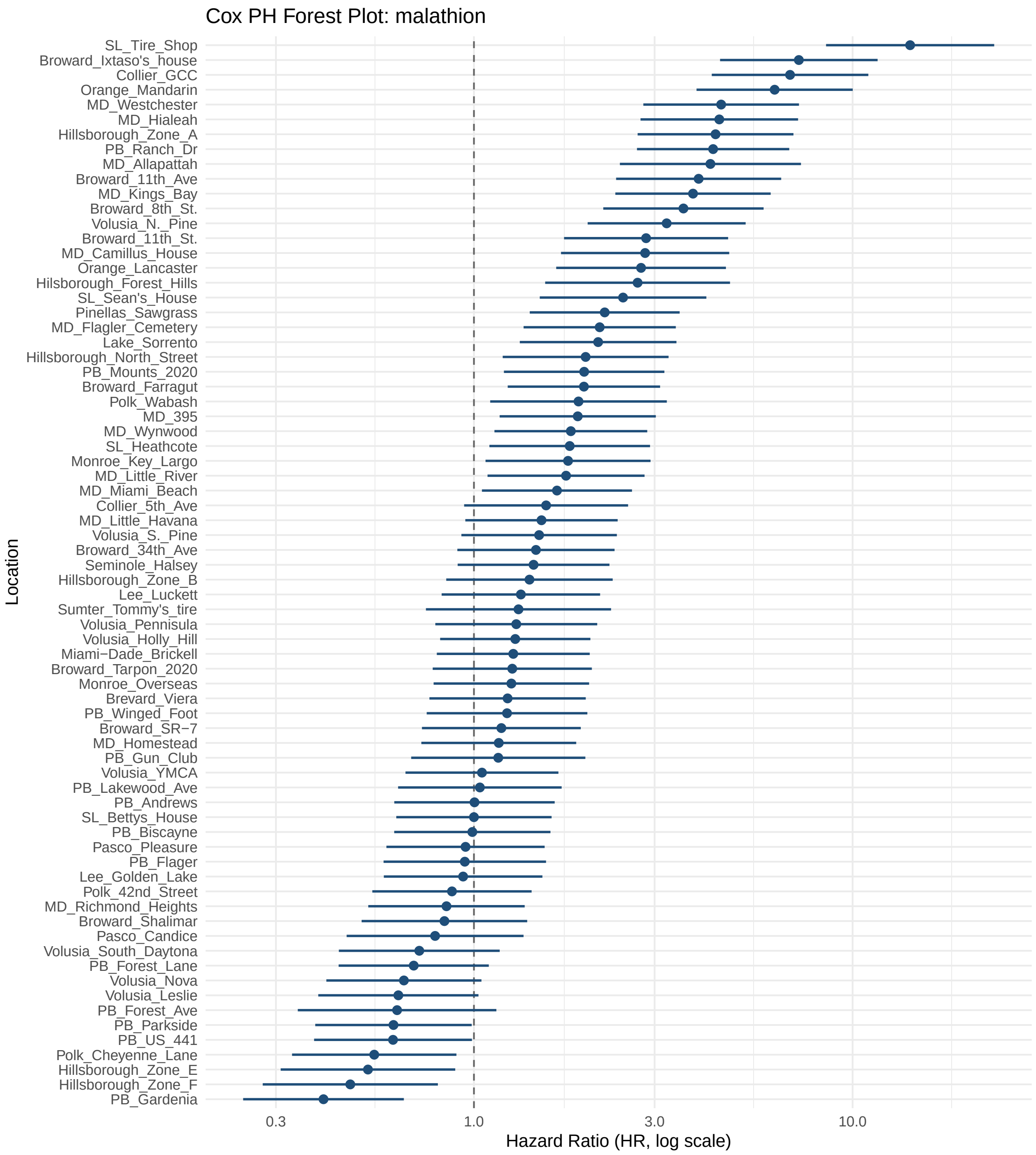

Kaplan-Meier Plot

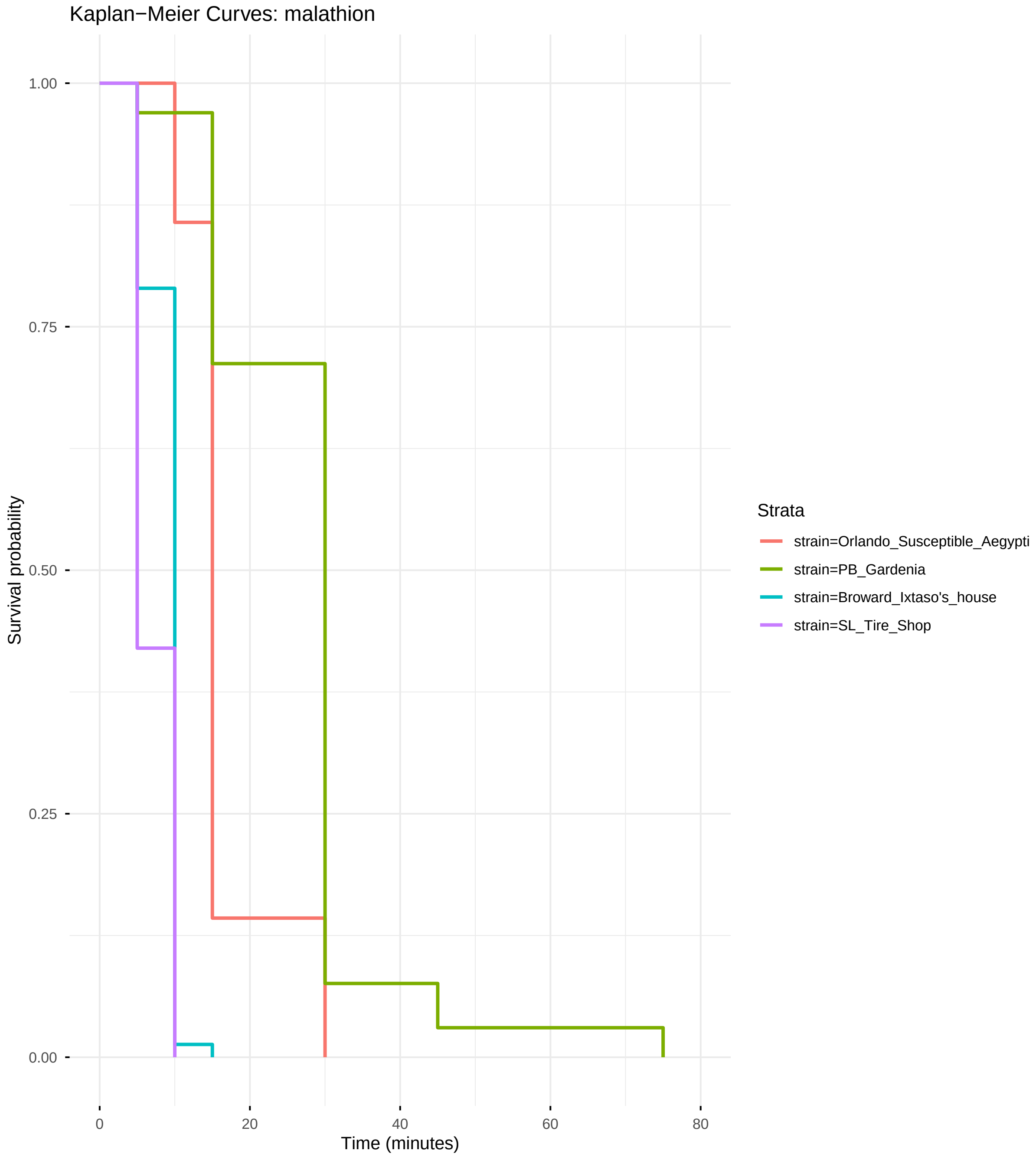

Naled

Cox PH Regression Table

Term	log(HR)	HR	SE	z	p-value
Brevard_Viera	4.529	92.691	0.449	10.08	0.000
Broward_11th_Ave	-0.560	0.571	0.466	-1.20	0.229
Broward_34th_Ave	0.296	1.345	0.456	0.65	0.516
Broward_8th_St.	1.350	3.857	0.457	2.95	0.003
Broward_Farragut	1.002	2.723	0.452	2.21	0.027
Broward_Ixtaso's_house	2.265	9.632	0.455	4.98	0.000
Broward_Shalimar	0.470	1.600	0.458	1.03	0.305
Broward_SR-7	3.697	40.339	0.455	8.12	0.000
Broward_Tarpon_2020	3.746	42.331	0.459	8.16	0.000
Collier_5th_Ave	4.175	65.025	0.459	9.10	0.000
Collier_GCC	-1.913	0.148	0.468	-4.08	0.000
Hillsborough_Forest_Hills	1.731	5.646	0.603	2.87	0.004
Hillsborough_North_Street	0.219	1.245	0.466	0.47	0.638
Hillsborough_Zone_A	0.948	2.580	0.452	2.09	0.036
Hillsborough_Zone_B	0.071	1.073	0.460	0.15	0.878
Hillsborough_Zone_E	-1.256	0.285	0.457	-2.75	0.006
Hillsborough_Zone_F	-1.748	0.174	0.472	-3.70	0.000
Lake_Sorrento	-0.303	0.738	0.459	-0.66	0.509
Lee_Golden_Lake	2.526	12.506	0.457	5.53	0.000
Lee_Luckett	0.993	2.699	0.462	2.15	0.032
MD_395	1.181	3.259	0.456	2.59	0.010
MD_Camillus_House	0.910	2.485	0.459	1.98	0.047
MD_Hialeah	3.158	23.535	0.473	6.67	0.000
MD_Little_River	1.259	3.521	0.453	2.78	0.005
MD_Richmond_Heights	-0.469	0.625	0.470	-1.00	0.318
MD_Westchester	0.398	1.489	0.461	0.86	0.389
Monroe_Key_Largo	2.751	15.653	0.455	6.05	0.000
Monroe_Overseas	4.357	78.038	0.455	9.58	0.000
Orange_Lancaster	2.026	7.582	0.460	4.40	0.000
Orange_Mandarin	2.287	9.843	0.450	5.09	0.000
Pasco_Candice	0.412	1.511	0.500	0.82	0.410
Pasco_Pleasure	0.643	1.903	0.454	1.42	0.157
PB_Andrews	-1.382	0.251	0.462	-2.99	0.003
PB_Biscayne	-0.200	0.819	0.459	-0.44	0.663
PB_Flager	0.721	2.057	0.456	1.58	0.114
PB_Forest_Ave	1.265	3.542	0.452	2.80	0.005

Term	log(HR)	HR	SE	z	p-value
PB__Forest__Lane	-1.498	0.224	0.474	-3.16	0.002
PB__Gardenia	0.933	2.541	0.459	2.03	0.042
PB__Gun__Club	-0.321	0.725	0.473	-0.68	0.497
PB__Lakewood__Ave	-0.672	0.511	0.467	-1.44	0.151
PB__Parkside	1.188	3.282	0.453	2.62	0.009
PB__Ranch__Dr	-0.036	0.965	0.459	-0.08	0.938
PB__US__441	-0.250	0.778	0.461	-0.54	0.587
PB__Winged__Foot	0.305	1.357	0.455	0.67	0.502
Pinellas__Sawgrass	-1.496	0.224	0.451	-3.32	0.001
Polk__42nd__Street	3.201	24.561	0.457	7.00	0.000
Polk__Cheyenne__Lane	1.077	2.935	0.458	2.35	0.019
Polk__Wabash	2.680	14.579	0.458	5.85	0.000
SL__Bettys__House	-0.156	0.856	0.453	-0.34	0.731
SL__Sean's__House	0.833	2.301	0.456	1.83	0.068
SL__Tire__Shop	0.296	1.345	0.461	0.64	0.521
Volusia__Leslie	0.831	2.295	0.455	1.83	0.068
Volusia__N.__Pine	-0.536	0.585	0.469	-1.14	0.253
Volusia__Nova	-1.388	0.250	0.451	-3.08	0.002
Volusia__Pennisula	0.498	1.645	0.462	1.08	0.281
Volusia__S.__Pine	0.307	1.360	0.459	0.67	0.503
Volusia__YMCA	-1.077	0.340	0.453	-2.38	0.017

Cox PH Forest Plot

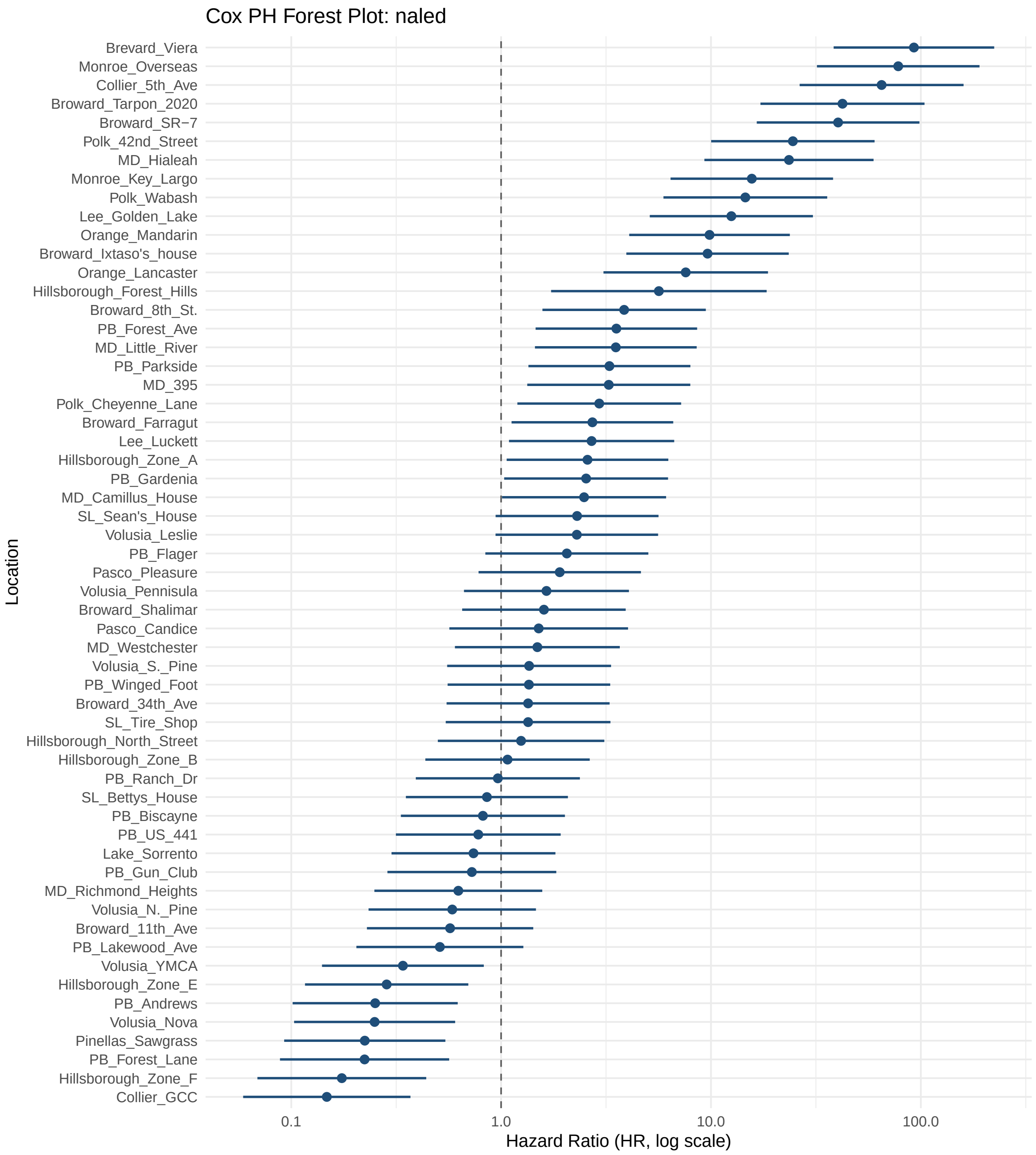

Kaplan-Meier Plot

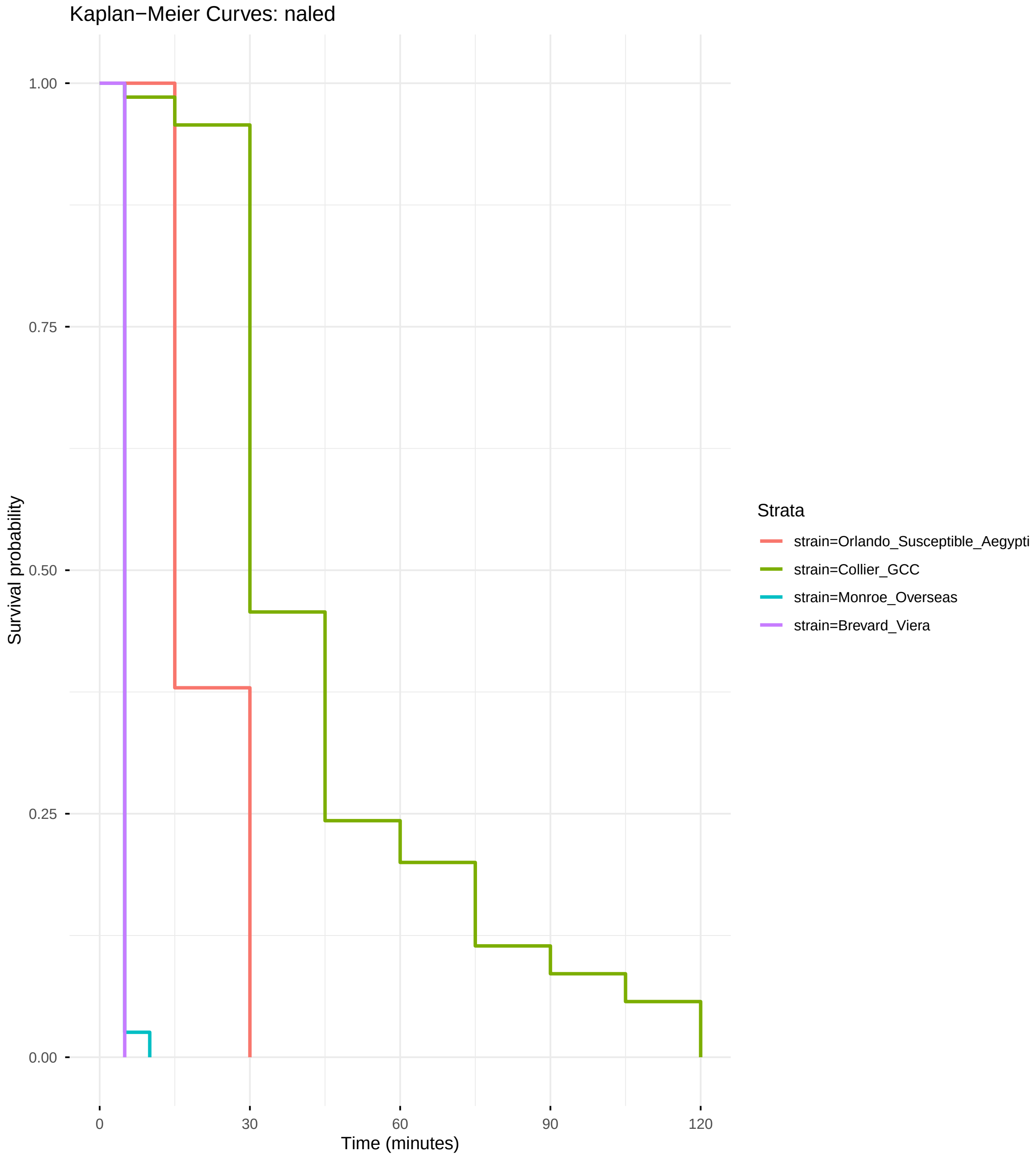

Permethrin

Cox PH Regression Table

Term	log(HR)	HR	SE	z	p-value
Brevard_Viera	-3.075	0.046	0.280	-10.97	0.000
Broward_11th_Ave	-3.077	0.046	0.301	-10.21	0.000
Broward_11th_St.	-0.817	0.442	0.285	-2.87	0.004
Broward_34th_Ave	-1.822	0.162	0.275	-6.62	0.000
Broward_8th_St.	-1.410	0.244	0.293	-4.81	0.000
Broward_Farragut	-2.455	0.086	0.274	-8.96	0.000
Broward_Ixtaso's_house	-1.597	0.203	0.272	-5.87	0.000
Broward_Shalimar	-2.406	0.090	0.291	-8.26	0.000
Broward_SR-7	-2.259	0.104	0.277	-8.14	0.000
Broward_Tarpon_2020	-1.933	0.145	0.298	-6.49	0.000
Broward_Tarpon_2022	-2.590	0.075	0.277	-9.34	0.000
Collier_5th_Ave	-2.411	0.090	0.275	-8.78	0.000
Collier_Corporate_Square	-1.508	0.221	0.274	-5.50	0.000
Collier_GCC	-1.370	0.254	0.285	-4.81	0.000
Hernando_Gupton	-1.935	0.144	0.296	-6.53	0.000
Hillsborough_Forest_Hills	-2.604	0.074	0.294	-8.87	0.000
Hillsborough_North_Street	-1.934	0.145	0.360	-5.37	0.000
Hillsborough_Zone_A	-0.881	0.414	0.278	-3.17	0.002
Hillsborough_Zone_B	-1.834	0.160	0.293	-6.27	0.000
Hillsborough_Zone_D	0.148	1.159	0.295	0.50	0.617
Hillsborough_Zone_E	-2.384	0.092	0.299	-7.97	0.000
Hillsborough_Zone_F	-2.073	0.126	0.301	-6.90	0.000
Lake_Sorrento	-1.569	0.208	0.289	-5.43	0.000
Lee_Golden_Lake	-1.661	0.190	0.273	-6.08	0.000
Lee_Luckett	-2.124	0.120	0.275	-7.72	0.000
MD_395	-2.693	0.068	0.285	-9.46	0.000
MD_Allapattah	-2.025	0.132	0.291	-6.95	0.000
MD_Brickell	-2.601	0.074	0.274	-9.49	0.000
MD_Camillus_House	-1.608	0.200	0.299	-5.38	0.000
MD_Flagler_Cemetery	-1.703	0.182	0.283	-6.01	0.000
MD_Hialeah	-2.231	0.107	0.287	-7.78	0.000
MD_Homestead	-2.319	0.098	0.277	-8.37	0.000
MD_Kings_Bay	-1.697	0.183	0.275	-6.16	0.000
MD_Little_Havana	-2.155	0.116	0.277	-7.79	0.000
MD_Little_River	-1.739	0.176	0.278	-6.24	0.000
MD_Miami_Beach	0.111	1.118	0.281	0.40	0.692

Term	log(HR)	HR	SE	z	p-value
MD_Richmond_Heights	-2.546	0.078	0.297	-8.56	0.000
MD_Westchester	-1.288	0.276	0.288	-4.47	0.000
MD_Wynwood	-2.782	0.062	0.270	-10.29	0.000
Monroe_Key_Largo	-1.830	0.160	0.272	-6.74	0.000
Monroe_Overseas	-1.832	0.160	0.273	-6.71	0.000
Orange_Howell	-2.375	0.093	0.298	-7.96	0.000
Orange_Lancaster	-3.153	0.043	0.302	-10.43	0.000
Orange_Mandarin	-1.085	0.338	0.284	-3.82	0.000
Pasco_Candice	-1.027	0.358	0.303	-3.39	0.001
Pasco_Pleasure	-2.486	0.083	0.273	-9.10	0.000
PB_Andrews	-2.924	0.054	0.306	-9.57	0.000
PB_Biscayne	-1.746	0.174	0.287	-6.08	0.000
PB_Flager	-2.172	0.114	0.283	-7.66	0.000
PB_Forest_Ave	-0.565	0.569	0.288	-1.96	0.050
PB_Forest_Lane	-1.635	0.195	0.281	-5.81	0.000
PB_Gardenia	-2.870	0.057	0.283	-10.14	0.000
PB_Gun_Club	-1.705	0.182	0.284	-6.01	0.000
PB_Lakewood_Ave	-1.912	0.148	0.291	-6.57	0.000
PB_Mounts_2020	-1.653	0.191	0.274	-6.04	0.000
PB_Parkside	-1.809	0.164	0.280	-6.45	0.000
PB_Ranch_Dr	-0.771	0.463	0.273	-2.83	0.005
PB_US_441	-0.796	0.451	0.285	-2.79	0.005
PB_Winged_Foot	-2.389	0.092	0.280	-8.54	0.000
Pinellas_Sawgrass	-2.842	0.058	0.274	-10.38	0.000
Polk_42nd_Street	-1.790	0.167	0.290	-6.18	0.000
Polk_Wabash	-2.593	0.075	0.290	-8.94	0.000
Seminole_Halsey	-1.901	0.149	0.272	-6.98	0.000
SL_Bettys_House	-2.433	0.088	0.279	-8.71	0.000
SL_Heathcote	-1.680	0.186	0.292	-5.74	0.000
SL_Sean's_House	-2.452	0.086	0.288	-8.51	0.000
SL_Tire_Shop	-1.378	0.252	0.288	-4.78	0.000
Sumter_Tommy's_tire	-2.253	0.105	0.313	-7.20	0.000
Volusia_Holly_Hill	0.204	1.227	0.269	0.76	0.447
Volusia_Leslie	-1.407	0.245	0.278	-5.06	0.000
Volusia_N._Pine	-3.223	0.040	0.301	-10.70	0.000
Volusia_Nova	-3.448	0.032	0.279	-12.37	0.000
Volusia_S._Pennisula	-1.966	0.140	0.284	-6.92	0.000
Volusia_S._Pine	-1.912	0.148	0.276	-6.92	0.000
Volusia_South_Daytona	-0.393	0.675	0.286	-1.37	0.170
Volusia_YMCA	-2.390	0.092	0.275	-8.68	0.000

Cox PH Forest Plot

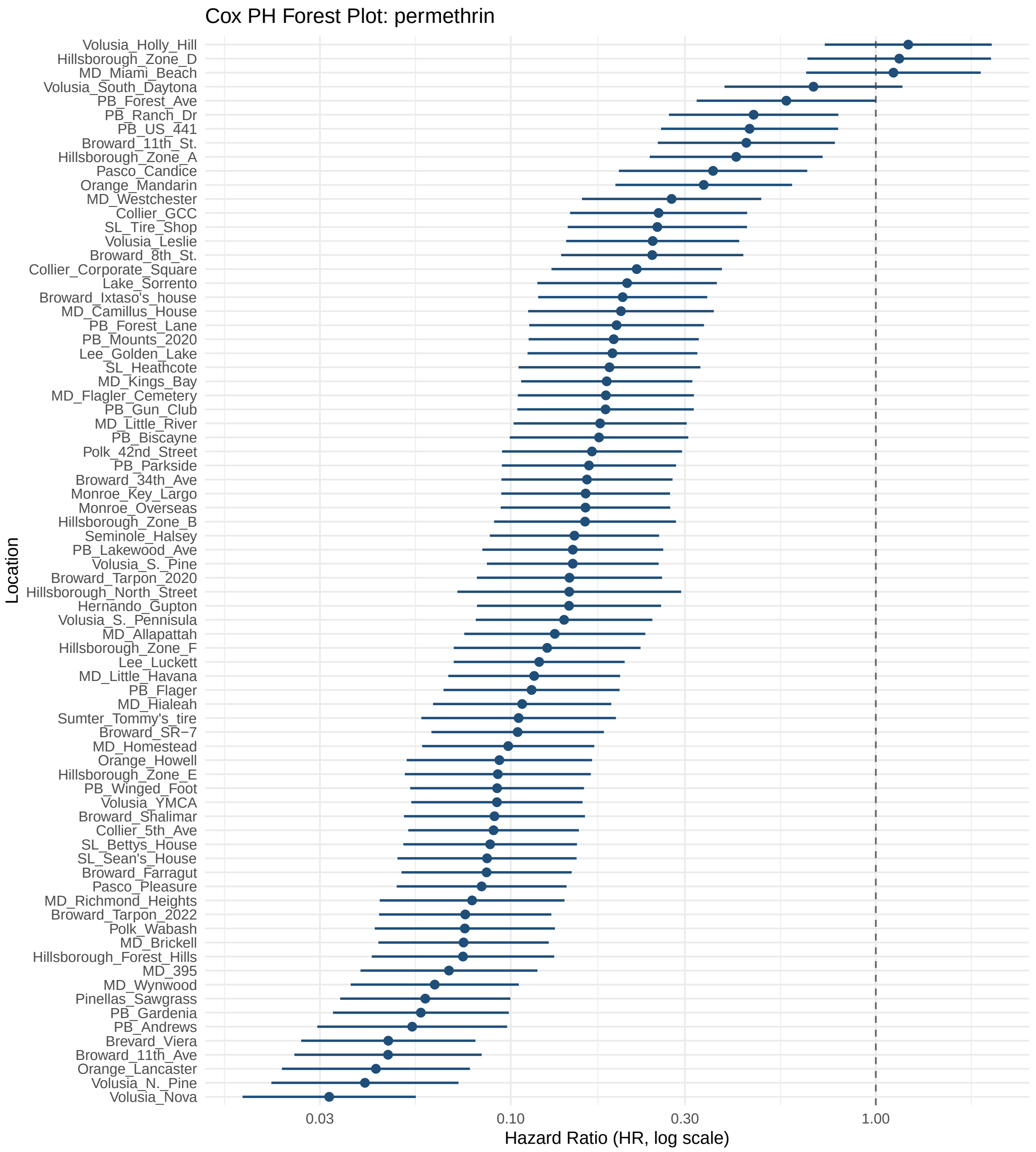

Kaplan-Meier Plot

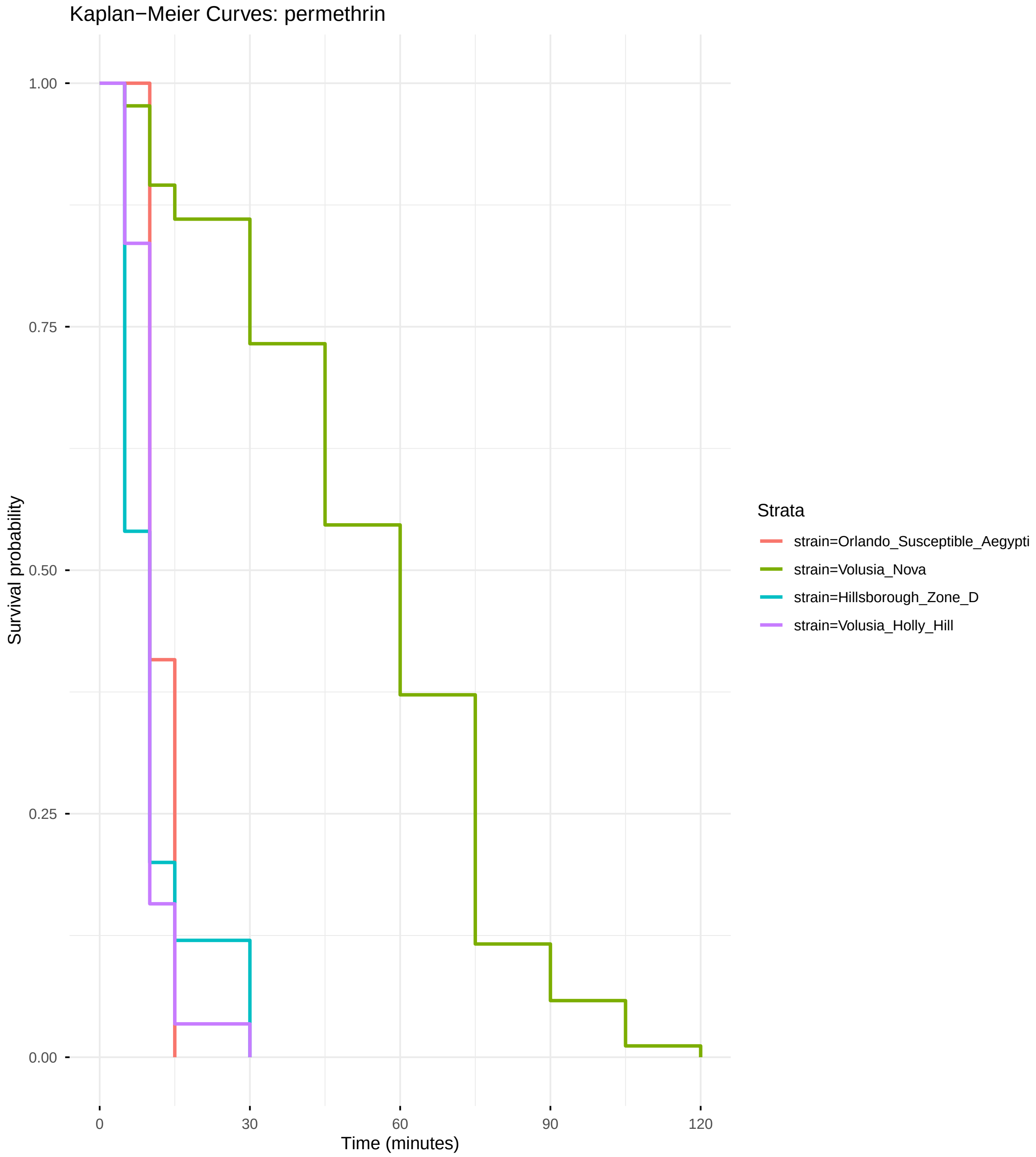

Sumithrin

Cox PH Regression Table

Term	log(HR)	HR	SE	z	p-value
Brevard_Viera	-4.284	0.014	0.282	-15.17	0.000
Broward_11th_Ave	-4.230	0.015	0.332	-12.75	0.000
Broward_11th_St.	-4.225	0.015	0.298	-14.16	0.000
Broward_34th_Ave	-4.072	0.017	0.275	-14.80	0.000
Broward_8th_St.	-3.627	0.027	0.279	-13.00	0.000
Broward_Farragut	-4.087	0.017	0.273	-14.97	0.000
Broward_Ixtaso's_house	-3.050	0.047	0.270	-11.30	0.000
Broward_Shalimar	-3.584	0.028	0.272	-13.20	0.000
Broward_SR-7	-3.993	0.018	0.259	-15.40	0.000
Broward_Tarpon_2020	-3.822	0.022	0.266	-14.37	0.000
Broward_Tarpon_2022	-3.801	0.022	0.269	-14.12	0.000
Collier_5th_Ave	-4.046	0.017	0.279	-14.52	0.000
Collier_Corporate_Square	-3.044	0.048	0.271	-11.24	0.000
Hernando_Gupton	-0.524	0.592	0.284	-1.85	0.065
Hillsborough_North_Street	-4.087	0.017	0.379	-10.80	0.000
Hillsborough_Zone_A	-3.822	0.022	0.298	-12.84	0.000
Hillsborough_Zone_B	-3.153	0.043	0.344	-9.17	0.000
Lake_Sorrento	-2.529	0.080	0.283	-8.93	0.000
Lee_Golden_Lake	-3.368	0.034	0.277	-12.14	0.000
Lee_Luckett	-3.387	0.034	0.271	-12.48	0.000
MD_395	-4.010	0.018	0.301	-13.34	0.000
MD_Camillus_House	-4.087	0.017	0.289	-14.15	0.000
MD_Flagler_Cemetery	-3.629	0.027	0.268	-13.52	0.000
MD_Hialeah	-3.631	0.026	0.286	-12.68	0.000
MD_Homestead	-4.000	0.018	0.268	-14.94	0.000
MD_Kings_Bay	-4.355	0.013	0.278	-15.65	0.000
MD_Little_Havana	-4.226	0.015	0.267	-15.85	0.000
MD_Little_River	-2.408	0.090	0.260	-9.26	0.000
MD_Miami_Beach	-4.457	0.012	0.280	-15.92	0.000
MD_Richmond_Heights	-4.067	0.017	0.279	-14.59	0.000
MD_Wynwood	-3.809	0.022	0.262	-14.53	0.000
Monroe_Key_Largo	-3.367	0.035	0.264	-12.76	0.000
Monroe_Overseas	-3.914	0.020	0.277	-14.12	0.000
Orange_Lancaster	-2.063	0.127	0.285	-7.24	0.000
Orange_Mandarin	-2.239	0.107	0.278	-8.04	0.000
Pasco_Candice	-3.523	0.030	0.286	-12.31	0.000

Term	log(HR)	HR	SE	z	p-value
Pasco_Pleasure	-4.076	0.017	0.268	-15.20	0.000
PB_Andrews	-4.646	0.010	0.292	-15.91	0.000
PB_Flager	-3.483	0.031	0.271	-12.84	0.000
PB_Forest	-4.947	0.007	0.416	-11.88	0.000
PB_Forest_Lane	-2.932	0.053	0.289	-10.15	0.000
PB_Gardenia	-3.300	0.037	0.276	-11.95	0.000
PB_Lakewood_Ave	-3.514	0.030	0.276	-12.72	0.000
PB_Mounts_2020	-3.740	0.024	0.264	-14.15	0.000
PB_Parkside	-4.347	0.013	0.643	-6.76	0.000
PB_Ranch_Dr	-1.925	0.146	0.267	-7.21	0.000
PB_US_441	0.226	1.254	0.292	0.78	0.437
PB_Winged_Foot	-3.743	0.024	0.286	-13.09	0.000
Pinellas_Sawgrass	-3.863	0.021	0.266	-14.51	0.000
Polk_42nd_Street	-2.180	0.113	0.265	-8.24	0.000
Polk_Cheyenne_Lane	-4.201	0.015	0.284	-14.80	0.000
Polk_Wabash	-4.593	0.010	0.318	-14.46	0.000
Seminole_Halsey	-2.686	0.068	0.262	-10.27	0.000
SL_Bettys_House	-3.960	0.019	0.281	-14.08	0.000
SL_Heathcote	-4.204	0.015	0.322	-13.06	0.000
SL_Sean's_House	-3.301	0.037	0.279	-11.82	0.000
SL_Tire_Shop	-4.179	0.015	0.299	-13.96	0.000
Volusia_Holly_Hill	-3.390	0.034	0.262	-12.93	0.000
Volusia_Leslie	-3.338	0.036	0.273	-12.23	0.000
Volusia_N._Pine	-4.323	0.013	0.286	-15.11	0.000
Volusia_Nova	-4.580	0.010	0.305	-15.01	0.000
Volusia_Pennisula	-3.985	0.019	0.308	-12.94	0.000
Volusia_YMCA	-3.620	0.027	0.267	-13.56	0.000

Cox PH Forest Plot

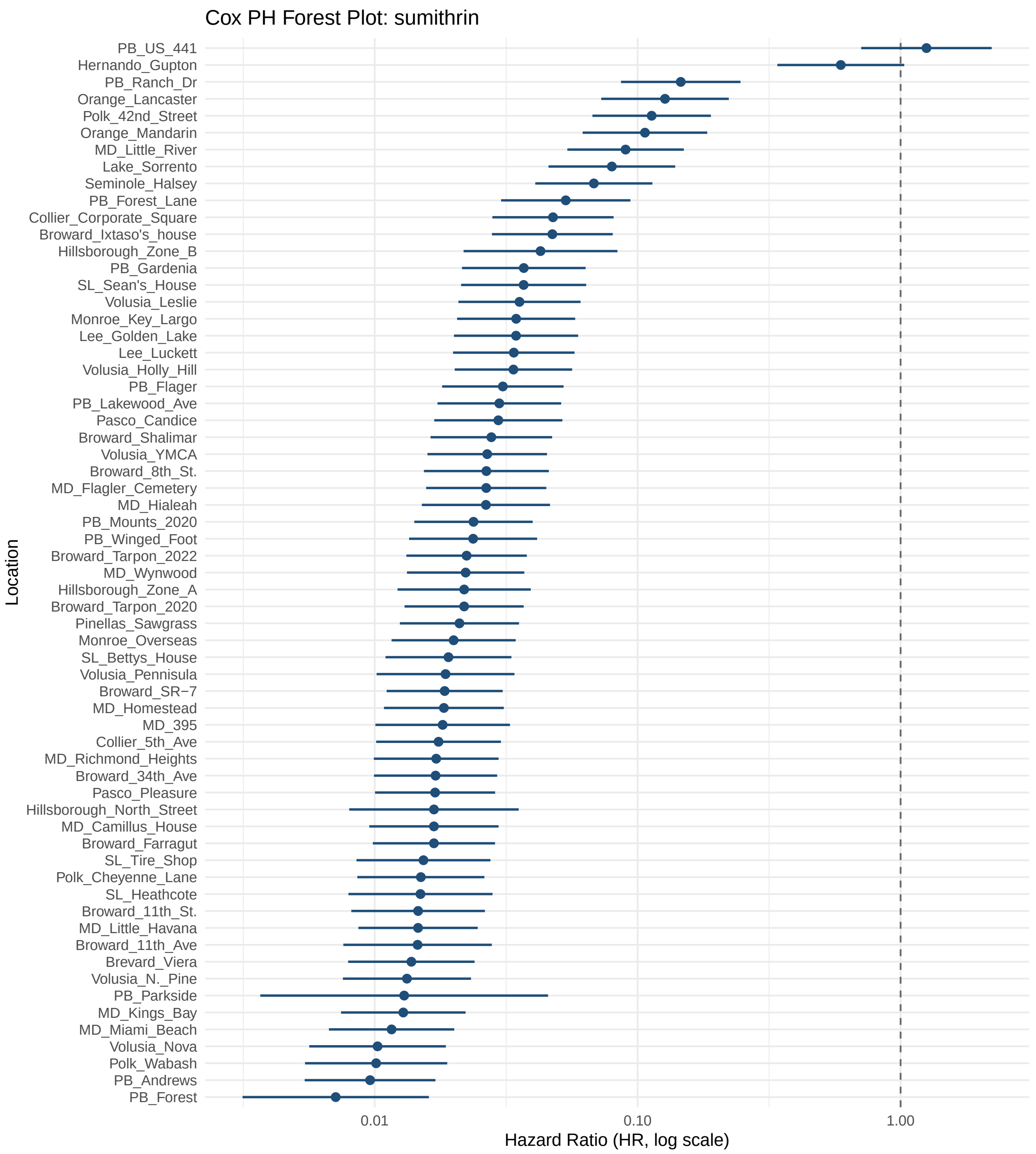

Kaplan-Meier Plot

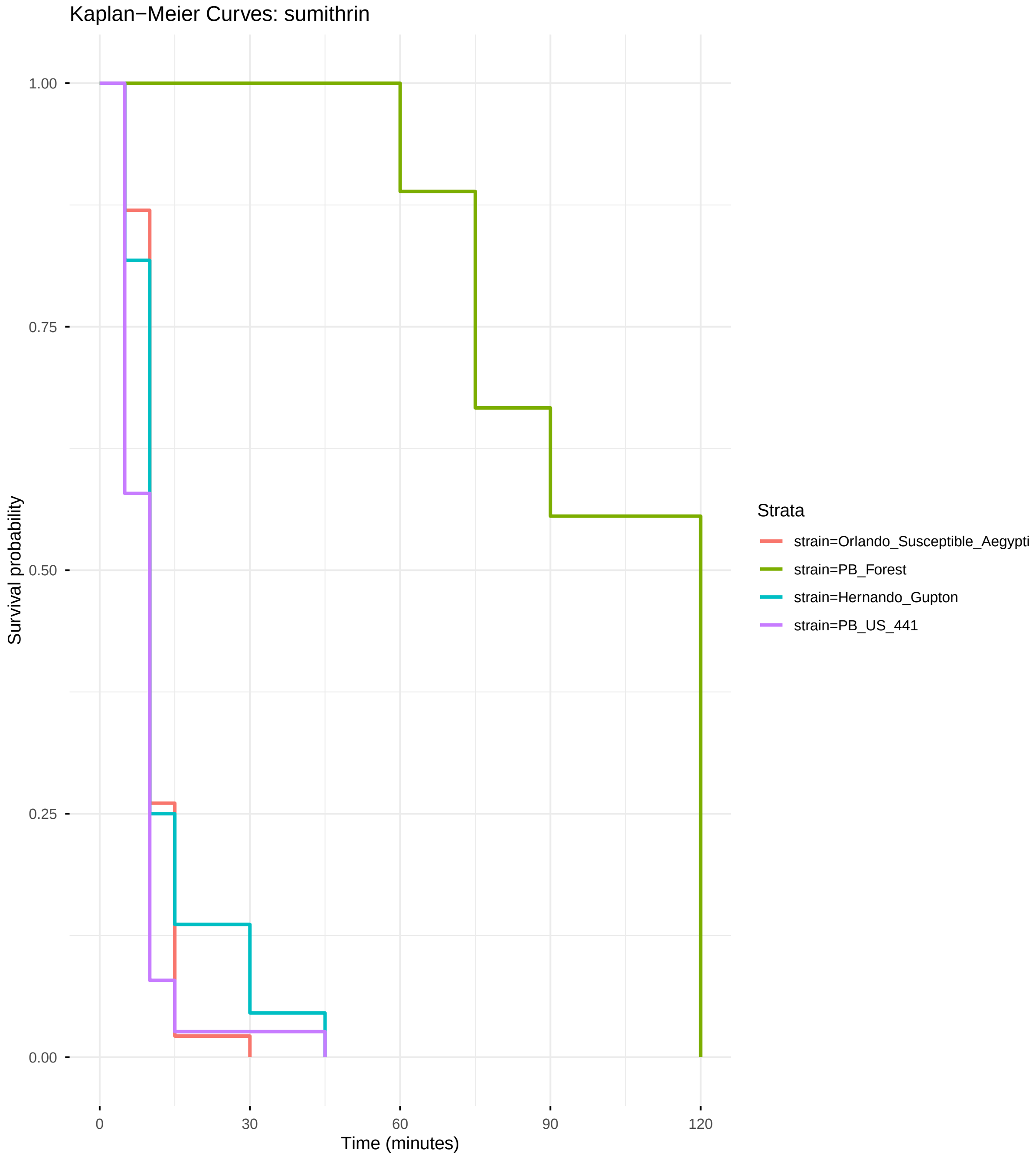

Supplement: Supplementary file 1 [file pathogens-15-00251-s001.zip › File S2.pdf]
